# Targeted DNA Demethylation: Vectors, Effectors and Perspectives

**DOI:** 10.3390/biomedicines11051334

**Published:** 2023-04-30

**Authors:** Naohiro Yano, Alexey V. Fedulov

**Affiliations:** Department of Surgery, Rhode Island Hospital, Alpert Medical School of Brown University, 593 Eddy Street, Providence, RI 02903, USA

**Keywords:** DNA demethylase, DNA-binding domains, gene targeting, epigenetic therapy, vectors

## Abstract

Aberrant DNA hypermethylation at regulatory cis-elements of particular genes is seen in a plethora of pathological conditions including cardiovascular, neurological, immunological, gastrointestinal and renal diseases, as well as in cancer, diabetes and others. Thus, approaches for experimental and therapeutic DNA demethylation have a great potential to demonstrate mechanistic importance, and even causality of epigenetic alterations, and may open novel avenues to epigenetic cures. However, existing methods based on DNA methyltransferase inhibitors that elicit genome-wide demethylation are not suitable for treatment of diseases with specific epimutations and provide a limited experimental value. Therefore, gene-specific epigenetic editing is a critical approach for epigenetic re-activation of silenced genes. Site-specific demethylation can be achieved by utilizing sequence-dependent DNA-binding molecules such as zinc finger protein array (ZFA), transcription activator-like effector (TALE) and clustered regularly interspaced short palindromic repeat-associated dead Cas9 (CRISPR/dCas9). Synthetic proteins, where these DNA-binding domains are fused with the DNA demethylases such as ten-eleven translocation (Tet) and thymine DNA glycosylase (TDG) enzymes, successfully induced or enhanced transcriptional responsiveness at targeted loci. However, a number of challenges, including the dependence on transgenesis for delivery of the fusion constructs, remain issues to be solved. In this review, we detail current and potential approaches to gene-specific DNA demethylation as a novel epigenetic editing-based therapeutic strategy.

## 1. Introduction

The exciting paradigm of epigenetics, that ‘genes are not your destiny’, has taken a novel turn with the development of targeted molecular tools to selectively modulate the epigenetic status (e.g., promoter methylation) and thus transcription of genes. The ability to take control over the epigenome and ‘edit’ it for therapeutic and experimental benefit represents an enticing but complicated endeavor. This review focuses on the rapid progress of site-specific DNA demethylation, made possible by technological innovation since the 2010s, but also traces the footsteps of DNA demethylation research since its dawn in the 1980s. The article summarizes the current knowledge on the mechanisms of DNA demethylation and details how they are or can be exploited to re-activate epigenetically silenced genes. Going beyond an overview of ‘vectored’ delivery of epigenetic modifiers, the paper introduces the concept of vector-free epigenetically acting agents, with a view to future application in epigenetic medicine.

## 2. DNA Methylation as a Key Epigenetic Mechanism of Gene Function Control

DNA methylation that alters gene expression without a change in the DNA sequence is the most widely studied epigenetic mechanism involved in the pathogenesis of various diseases [1,2]. DNA methylation is a covalent modification that occurs to cytosines in the context of cytosine-phosphate-guanosine (CpG) residues; when it occurs in key regulatory regions of the gene (e.g., promoter), methylation suppresses the expression of the gene [3]. Virtually all cells in an organism contain all the genes of that organism’s genome; epigenetic mechanisms are broadly involved in the silencing of ‘unused’ genes which underlies the difference in structure and function of cells [4,5]. This silencing is a powerful and inheritable mechanism of gene function control [4]. DNA methylation is a key player in epigenetic silencing of transcription [3,6]; conversely, lowered methylation of promoters or regulatory intragenic regions has been linked to enhanced transcription [5,7,8]. DNA methylation of CpG dinucleotides is catalyzed by DNMT3a and b, and DNMT1 maintains the methylation during semiconservative DNA replication [9]. In case this maintenance methylation fails for some reason, e.g., inhibition, depletion or nuclear exclusion of DNMT1, so-called “passive demethylation” occurs in dividing cells [10]. Passive demethylation is a relatively slow process and theoretically does not occur in terminally differentiated non-dividing cells [11]. Given these points, it is unlikely that passive demethylation plays a leading role in demethylation in most adult somatic cells that have completed mitosis [12], although it plays an important role when genome wide DNMT inhibitors are employed therapeutically.

The first report on biological active DNA demethylation processes independent of cell replication in vertebrates was published in 1984 [13], in which HpaII site 611 base pairs upstream from the 5’ end of the of the chicken vitellogenin II gene were seen to exhibit estrogen-dependent demethylation independent of DNA synthesis, predicting the existence of a site-specific demethylase. However, no further characterization of this enzyme has been forthcoming. Numerous evidences of active demethylation have been reported since then [14,15,16,17,18]. Another common example of active demethylation is the demethylation of pluripotency genes in nuclear reprogramming. During normal development, genes related to pluripotency are constitutively silenced by histone and DNA methylation. For example, the proximal m5CpG of the oct4 gene regulatory site in thymocyte nuclei transplanted into Xenopus oocytes is immediately demethylated [19]. Demethylation usually occurs with changes in physiological cellular states. Examples include changes in cell differentiation, such as terminal differentiation, or when exposed to nuclear hormones, protein kinase C (PKC) activators, histone deacetylase inhibitors (HDACi) or changes in neural activity [20,21,22,23,24,25]. These findings indicate that active demethylation is a widespread phenomenon in both dividing and differentiated cells.

## 3. Etiological Significance of DNA Methylation

In mammals, about 60–80% of the CpG sites in the genome are modified into 5mC [7]. CpGs outside promoter CpG islands are usually highly methylated, while those within promoter CpG islands are mostly unmethylated [26,27]. However, in several diseases, the promoter CpG islands of genes ‘protective’ against the disease are found to be aberrantly methylated [28,29,30]. Epigenetic alterations in CpG islands and, more broadly, elsewhere across the epigenomic landscape have been increasingly reported, ranging from the most frequent examples, linking DNA hypermethylation to cellular carcinogenesis, to a plethora of cardiovascular, renal, hepatic, metabolic and infectious diseases and environmental exposures [31,32,33,34,35,36,37]. Thus, the scope of aberrant methylation in pathogenesis is becoming more and more elucidated. For example, aberrations of cell cycle checkpoints in cancer result in abnormal cell proliferation and chromosomal instability. Examples include abnormal methylation of the p53 gene [38,39,40], or hypermethylation of the checkpoint genes with Fork-head and Ring finger (CHFR) in cancer cells [41,42,43,44,45,46,47,48,49].

While methylation and transcription are usually inversely correlated, and this is typically true for promoter methylation, there are exceptional situations where an increase in methylation leads to an increase in transcription if this methylation change affects a key regulatory area of a special kind [50,51], which can happen with enhancers, or where increased methylation inhibits binding of repressive factors or methylation-sensitive transcription factors. In general, however, selective demethylation of genes occurs during gene expression enhancement, but it is not certain to what extent demethylation is a primary factor versus a secondary consequence of enhanced transcription.

Moreover, DNA demethylation may not always be sufficient to increase transcription. For example, demethylation of the IL2 promoter is necessary but not sufficient for gene activation [52]. In this case, binding of the transcription factor Oct-1 to the promoter-enhancer region of the IL2 gene is a higher priority requirement for enhanced gene expression. When Oct-1 binds to the promoter-enhancer region as a result of additional stimulation, the IL2 gene becomes more sensitive to DNA demethylation and its gene expression is more highly upregulated. When the stimulus is removed, Oct-1 remains in the promoter-enhanced region, making the second and subsequent induction of gene expression faster and stronger [52]. Thus, priming of the locus occurs to prepare for subsequent external stimuli. The transcriptional result of gene demethylation is in this case dependent on the presence of a background stimulus: if present, the newly demethylated gene becomes intrinsically upregulated, otherwise it will ‘wait’ for an extrinsic trigger. This leads to an important potential benefit of epigenetic editing over traditional gene overexpression or a recombinant protein administration: demethylation opens the promoter to situation-appropriate, context-dependent intrinsic (or extrinsic, if desired) stimuli.

Aberrant hypermethylation occurs as a result of intrinsic deregulation or upon exposure to environmental toxicants; many environmental exposures have been linked to epigenomic effects [53,54] leading to two major challenges in the field. First, while the epidemiologic data for association of exposures with epigenomic effects are strong, proof of causality for exposure-related methylation changes at specific loci has been impossible due to a lack of experimental tools to specifically revert these alterations. Second, there are no therapeutic agents that can gene-specifically reverse ‘harmful’ epigenetic changes and achieve epigenetic-based treatment. These challenges offer an attractive opportunity to employ DNA demethylation as a therapeutic strategy for diseases where DNA hypermethylation is involved. Until recently, only non-specific agents, such as cytidine analogs including 5-aza-2′-deoxycytidine, have been available for removing methyl groups from CpGs based on the inhibition of DNMTs, and they have been widely used to study the effects of demethylation on gene promoters [55,56,57,58]. However, these non-specific agents lead to global demethylation of CpGs, making it impossible to define the causal effects of specific CpG aberrations [59]. Following these experimental trials, active DNA demethylation by enzymatic activity has become the focus of attention as an experimental and potential future therapeutic strategy [60], promoting interest in gene-specific (or site-specific) demethylation approaches [61].

## 4. Biochemical Mechanisms of Active DNA Demethylation

It is increasingly recognized that active demethylation plays an important role in a wide variety of biological processes, and much attention and effort has been devoted to the elucidation of the mechanism of active demethylation, including the identification of the involved demethylase(s). DNA demethylase activity was noted for the first time in extracts of mouse erythroleukemia nuclei [62]. It was found that 5-methylcytosine (5mC) is finally replaced by C in a replication-independent manner, but this mechanism has not been elucidated in detail for a long time. Over the past years, several studies have proposed various possible mechanisms through which active DNA demethylation may take place. These include enzymatic removal of methyl groups at 5mC, a series of intermediary conversions, participation of the base excision repair (BER) and less well-known mechanisms.

### 4.1. Enzymatic Removal of the Methyl Group of 5mC

In the initial step of the envisioned DNA demethylation process, 5mC is converted to 5-hydroxymethyl cytosine (5hmC) by the addition of atomic oxygen; 5hmC is further oxidized to 5-formylcytosine (5fC) and finally 5-carboxylcytosine (5caC) [63]. This is an important step that allows for several alternative or duplicative methods to remake normal cytosine residues autonomously or in a DNA replication-dependent manner, ultimately removing the methyl mark [64,65,66]. The oxidized intermediates, 5fC and 5caC, are removed and can further regenerate unmethylated cytosine at the target site through the BER mechanism. The first half of the oxidation reactions are catalyzed by the ten-eleven translocation protein (TET) family enzymes, while the second half, removal of 5fC and 5caC, is catalyzed by thymine DNA glycosylase (TDG) [67].

The first member of TET family, TET1, was discovered in acute myeloid leukemia (AML) cells as a fusion partner for histone H3 Lys4 methyltransferase [68]. Subsequent in vitro studies have shown that the enzymatic activity of human TET1 includes the ability to hydrolyze 5mC [69]. TET protein can act on both fully methylated and hemimethylated DNA [70]. This ability of TETs to oxidize 5mC is particularly important in embryonic stem cells (ESCs), which must maintain the ability to self-renew and take on diverse 5mC patterns [71]. The TET protein is characterized by its distinctive core catalytic domain, and its isoforms are known to be cell type specific [72,73]. Mammals encode three TET protein orthologues (TET1, TET2, and TET3) [74]. The three TET proteins have similar C-terminal catalytic domain-containing cysteine-rich (Cys) regions and a double-stranded b-helix (DSBH) fold which exhibits 2-oxoglutarate (2-OG)- and Fe(II)-dependent dioxygenase activity [75].

### 4.2. BER Through Direct Excision of 5mC Converts

Following the sequential oxidation reactions described above, TDG initiates the removal of target bases to make abasic sites [76]. Subsequently, apyrimidinic acid (AP) lyase activity nicks the DNA backbone, producing 5′ phosphomonoesters and 3′ sugar phosphate residues. AP endonuclease then removes the 3′ sugar chain, leaving a single nucleotide gap that will eventually be filled by DNA repair polymerase or ligase [77]. Genetic and biochemical evidence suggested at first that this mechanism is used in plants [78], whereas evidence supporting the existence of similar mechanisms in the vertebrates was less compelling. In 2000, a study using extracts from chick embryos suggested that this repair mechanism may contribute to DNA demethylation in vertebrates as well [79]. This study also demonstrated the existence of 5mC glycosylase activity against hemimethylated DNA in the extract and revealed that the enzyme responsible for this activity is a homologue of human TDG. Therefore, the 5mC glycosylase activity detected in this chicken embryo extract was assumed to be due to TDG. However, the excision activity of TDG against 5mC is only 1/30 to 1/40 of that against T/G mismatch, and TDG does not have the activity to cleave N-glycosidic bonds [80]. Thus, the details of the involvement of TDG in DNA demethylation needed to be further explored. Since TDG is known to act on hydroxymethylated cytosines as well as 5fC and 5caC via its specific recognition site, though not on 5mC [80,81,82,83], it suggests a reasonable concept of the demethylation process that entails starting with a conversion of 5mC to hmC/fC/caC by TETs, followed by TDG action. This is confirmed by the observation that, in TDG-deleted cells, an accumulation of 5fC/5caC is observed [84]. Given that on average ~10% of mCs in mammals are hmCs (bisulfite-based methods, e.g., pyrosequencing, do not distinguish the two forms), this could explain the consistent but low-impact results of using TDG alone in epigenetic editing studies [85,86]. Indeed, low levels of active, replication-independent DNA demethylation were seen to be mediated by TDG [84].

The action of TDG as a demethylase, whether involving BER or not, is supported by epigenetic editing reports using solely this enzyme [85,86]. Assessment of the relative input of TETs and TDG in demethylation can be complicated by the intrinsic presence of either enzyme in the nucleus. This has been addressed in a convincing demonstration that TET and TDG interact with each other in several ways in the demethylation process: TET1 and TDG interact physically, forming stable TET1–TDG complexes [87]. Moreover, TET1CD and TDG act in concert to release 5mC so that the excision of 5mC and 5hmC from DNA requires the catalytic activities of both TET1 and TDG; finally, TET stabilizes TDG activity, and their coordinated action engages BER in a way that avoids double strand breaks [88]. Hence, TDG is not simply a sequential follower of TET, as was previously considered.

This information about TDG is not inconsistent with its previously presumed role as a demethylase following the deamination of mC by the AID (activation-induced deaminase)/APOBEC (apolipoprotein B mRNA-editing enzyme complex) family of cytidine deaminases [69,89]. Deamination-mediated demethylation could also involve the DNA damage response protein GADD45, or even methyl-binding domain protein MBD4 [19,90,91]. However, there is evidence that contradicts this premise [92,93]. It therefore appears that deaminases are unlikely to be enzymatic effectors for epigenetic editing.

### 4.3. Potential for Enzymatic Demethylation without Excision and BER

Studies in embryonic cells allow the derivation of a mechanism in which initial conversion of mC to hmC by TET is followed by passive dilution where, as a result of replication, the unmodified C is regenerated [94,95]. It remains unclear to what extent this occurs during epigenetic editing, but it is plausible in dividing cells. It has also been reported that DNMTs that normally methylate the cytosine can, in some conditions, have a reverse effect, directly transforming mC to C [96].

AlkB homologs (ALKBH) are the other class (together with TET) of the Fe(II)/2-Oxoglutarate (2OG)-dependent dioxygenase superfamily of enzymes that have demethylating activity, and unlike TET they are capable of directly demethylating some of their substrates [97]. Besides their action on 3mC and 4mC, ALKBH act on 5mC [98], but while the outcome of the reaction for 3mC is unmethylated C, for 5mC it stops at 5caC, which then likely becomes the substrate for TDG.

Limited evidence suggests that 5caC may be converted into C directly and without excision: it was shown that both bacterial and mammalian C5-MTases can catalyze the direct decarboxylation of 5caC (but not of 5DC), yielding unmodified cytosine in the DNA [99]. It is possible that future studies will elaborate further on active enzymatic DNA demethylation. The attractiveness of this or another direct enzymatic conversion mechanism that avoids base excision lies in the lower risk of strand breaks and potential mutagenesis, which may confound translational approaches in the future.

## 5. Biological Effects of De-Repression of Epigenetically Silenced Genes

De-repression of epigenetically silenced genes by active, targeted DNA demethylation can have direct, exciting potential applications in a number of experimental strategies and human diseases:

### 5.1. For Instructing Cells to Produce a Protective/Therapeutic Protein

One benefit of the re-activation of epigenetically silenced genes is to confer unusual (but desirable) property to cells, e.g., production of a protein that is not typically produced by a given cell type, for example, a cytokine or immunoregulatory molecule. As an example, targeted de-methylation of nitric oxide synthase silenced in fibroblasts [85,86] confers to them an ability to produce nitric oxide (NO), which is a key component of innate immunity, including direct anti-microbial effects [100,101,102,103]. Enhancement of NO production by upregulation of NOS2 expression is a desirable therapeutic strategy [86,104]; reactivation of the enzyme’s gene could lead to increased production at targeted tissue sites.

### 5.2. In Targeting Specific Monogenic Epigenetic Aberrations That Single-Handedly Cause Disease

For example, fragile X Syndrome is a form of autism linked to epigenetic silencing in the promoter of the FMR1 gene [105]; demethylation of its promoter could restore transcription, and this presumption has recently been clearly demonstrated [106].

To modify expression of cancer-related genes: silencing of tumor-suppressor promoters by DNA hypermethylation is an important mechanism of carcinogenesis [107] and could be selectively targeted.

### 5.3. In Experimental Strategies Such as Transdifferentiation and Regeneration

#### 5.3.1. Transdifferentiation

Artificial overexpression of non-typical genes in fibroblasts transforms these cells into other types [108,109]; epigenetic editing could provide heritable effect without the need for transgenesis.

#### 5.3.2. Proliferation

Specialized cells (cardiomyocytes or neurons) are non-dividing because genes responsible for cell division are switched off; epigenetic editing of cell cycle regulators could drive DNA synthesis and potentially lead to proliferation [110].

However, until recently, most of the identified DNA methylation changes are used either correlatively, or as biomarkers for various diseases: 1. diagnostic markers [111]; 2. prognostic markers [112,113]; 3. markers for optimal treatment according to disease subclasses [114]; 4. markers for monitoring treatment efficacy [115]; 5. markers to identify genes to be examined for the development of epigenetically targeted therapies [107]. These DNA hypermethylated markers have been studied most extensively in cancer [116]. However, aberrant DNA hypermethylation is being implicated in other diseases. A selection of recently published reports on non-cancer disease-linked promoter DNA hypermethylation is shown in Table 1. As shown, gene promoters are abnormally hypermethylated in many diseases, making them prime candidates for therapeutic targets. Therefore, developing strategies for site-specific CpG promoter demethylation of disease-associated genes is a critical and exciting challenge.

## 6. Gene-Specific DNA Demethylation

Site-specific gene targeting DNA demethylation is accomplished by fusing the demethylases to engineered DNA-binding domains (DBDs) that tether the enzyme to the desired DNA sequence [61]. Since it was first reported simultaneously by three independent groups [85,86,141,142], the development of this innovative technology has opened the possibility of epigenetic therapeutic applications for various diseases. Here, we review the latest findings on strategies for site-specific DNA demethylation and discuss the prospects for clinical translation in the future.

### 6.1. Selection of Demethylase for Targeted DNA Demethylation

The most frequently reported DNA demethylase for gene targeting are the TET family proteins. Among these, TET1 fusion protein is most commonly used. However, there are scattered reports using TET2 and TET3, which are considered to have enzymatic activity equivalent to that of TET1, at least in vitro [75], although not in all experimental settings [141].

An increasing number of studies have reported the success of tethering TETs to DNA for targeted demethylation in recent years [143,144,145,146,147]. In the context of T cell differentiation, the dCas9-TET1catalytic domain (CD) demethylates the FOXP3-Treg specific demethylated region (TSDR) of Jurkat cells, leading to a Treg-like phenotype, suggested the feasibility of dCas9-TET1-mediated Treg programmation of primary T cells [148,149]. 

In cancer cells, dCas9-TET1 enhances the cisplatin sensitivity of A549 cells (lung carcinoma epithelial cells) by promoting the expression of nicotinamide nucleotide transhydrogenase (NNT) [150]. dCas9-TET1CD was used to demethylate the EphA7 gene: the protein correlates with life expectancy in cervical cancer, indicating the potential of dCas9-TET1CD as a therapeutic strategy for cervical cancer [151]. Leucine-rich repeat and immunoglobulin-like domain (LRIG) 1 is a negative regulator of receptor tyrosine kinases and a tumor suppressor; decreased LRIG1 expression is consistently observed in various types of cancer and is linked to poor patient prognosis [152]. dCas9-TET1CD-mediated demethylation along with VP64-mediated transcriptional activation increased endogenous LRIG1 expression in breast cancer cells, and reduced cancer cell viability [153]. TET1CD reactivated X-linked endogenous FOXP3 in breast cancer [154]. The tripartite-motif (TRIM) family proteins contribute to cancer initiation, progress, or therapy resistance, exhibiting tumor-suppressive functions: they are frequently downregulated by promoter methylation in cancerous tissues [155]. dCas9-TET1CD induced specific demethylation of TRIM58 in renal carcinoma cells (RCC) [156]. Promoter of the TMEM244 gene was demethylated to upregulate its expression in Sézary syndrome—an aggressive form of cutaneous T-cell lymphoma [157]. dCas9-TET1CD could demethylate hepatocyte nuclear factor (HNF)1A and Beta-1,4-mannosyl-glycoprotein 4-beta-N-acetylglucosaminyltransferase (MGAT3) genes in BG1 (human ovarian adenocarcinoma) cells [158], and telomeric repeat-containing RNA (TERRA) in HeLa (human cervix carcinoma) and T98G (human glioblastoma) cell lines [159].

The following reports have been published on the function of TET1 in non-tumor cells. TET1 ablation impairs cardiac differentiation of mouse embryonic stem cells and re-expression of the TET1CD rescued the differentiation defect in Tet-triple knockout mESCs [160]. Cyclin-dependent kinase-like (CDKL) 5 is associated with X-linked infantile spasm syndrome (ISSX). Halmai et al. performed artificial X chromosome inactivation (XCI) using dCas9-TET1CD targeting CDLK5 [161]. Rett syndrome is an X-linked neurodevelopmental disorder caused by loss-of-function heterozygous mutations of methyl CpG-binding protein 2 (MECP2) on the X chromosome in young females [162]. Qian et al. reported reactivation of MECP2 in human embryonic stem cells (hESCs) derived from RTT by dCas9-TET1CD [163]. These two reports suggest the potential of dCas9-TET1CD for therapeutic application in X chromosome associated diseases. dCas9-TET1CD also could re-activate Oct4 in NIH-3T3 cells [164] and beta-galactoside alpha-2,6-sialyltransferase 1 (ST6GAL1) in CHO cells [165].

A few studies signal readiness to transition epigenetic editing experiments to in vivo. Fragile X syndrome (FXS), the most common genetic form of intellectual disability in males, is caused by silencing of the FMR1 gene associated with hypermethylation in the CGG expansion mutation in the 5′ UTR of FMR1. dCas9-TET1CD was used to demethylate the CGG repeats in the brain cells of mice so that FMR1 expression in edited neurons was maintained in vivo after engrafting into the mouse brain [106]. With dCas9-TET1CD system and hydrodynamic tail vein injection, Hanzawa et al. showed targeted DNA demethylation of the Fgf 21 promoter in the liver of PPARα-deficient mice [166]. Russell–Silver syndrome (RSS) is a rare disorder characterized by intrauterine growth restriction (IUGR) and poor growth after birth. The patients of RSS show H19 upregulation and insulin-like growth factor (Igf) 2 downregulation. Horii et al. developed RSS model mice via demethylated H19 promotor lesion with embryonic microinjection of dCas9-TET1CD [167]. Noack et al. delivered dCas9-TET1CD targeted Dchs1, a regulator of corticogenesis, to mouse brains by electroporation and verified the biological relevance of the aberrant methylation of Dchs1 in developmental malformations and cognitive impairment [168].

On the other hand, TDG alone has been successful in demethylating and transcriptionally enhancing the NOS2 gene [85,86]. Furthermore, targeted DNA demethylation with Release of Silencing 1 (ROS1) 5mC DNA glycosylase [169] fused to the DNA-binding domain of yeast GAL4 (GBD) has been reported [170]. These reports are supplemented by studies using targeted fusions with P300 acetyltransferase, which helps support the epigenetic editing effect on gene transcription [149,171]. Thus, the selection of demethylases may be broadened in the future.

Given the multi-step nature of transformations from mC to C where TET proteins and TDG glycosylases interact in moving the atoms of the CH3 group, the strategies for application of multiple demethylases for epigenetic editing form a novel area of studies that may be called ‘atomic epigenetics’. When interpreting the reports of each single enzyme successfully employed for demethylation, it is important to consider that the other enzymes were intrinsically present as is natural for unmodified cells. At the same time, the paucity of studies on combinatorial use of different demethylases presents a space for future investigations.

### 6.2. Site-Specific DNA-Binding Domains

There are several types of DBDs that have been fused with the putative demethylases to tether them sequence-specifically to the promoters of interest. Aside from customizable DBDs aimed to allow single gene specificity, other naturally occurring DBDs have historically been the first used to affect a group of genes at once. For example, the Rel-homology domain (RHD) of NF-kB has been employed to target TDG to all NF-kB-dependent genes [85,86]. This and similar systems (using for example transcription factor binding domains) may be employed in epigenetic editing for re-activation of intrinsic transcription using a ‘grouped’ approach.

Recently, more specific and customizable DBDs have become applied. One of these is the recombinant transcription activator-like effector (TALE) [142,172]. TALE is a class of naturally occurring DNA-binding proteins found in the plant pathogen Xanthomonas sp. The DNA-binding domain of each TALE consists of 34-amino acid repeat modules arranged in tandem that can be rearranged according to a simple cipher to target new DNA sequences. Customized TALEs can be used for a wide variety of genome engineering applications, including transcriptional modulation and genome editing [173]. The molecular tool through which DNA demethylase is targeted to the disease-related gene promoters can be made by tethering human TET1 demethylase enzyme to a TALE repeat that has targeted DNA-binding specificities [142]. Modified TALE repeats provide an attractive platform for guiding TET1 activity. This is because assembly of individual repeat domains of known nucleotides can be used to produce large amounts of monomeric proteins that bind to virtually any target DNA sequence [174]. However, TALEs may be less suitable specifically for de-methylation, as they have difficulty binding richly methylated sequences [175].

**Figure 1:**
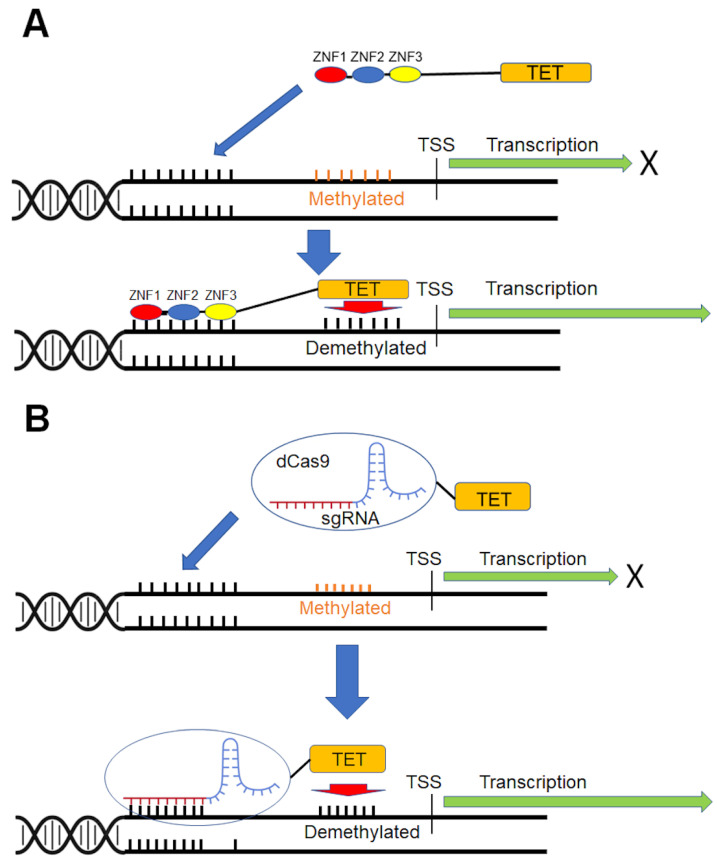
ZFA-TETs fusion protein (**A**) and CRISPR-dCas9-TET based (**B**) site-specific demethylation.

Concomitant to the TALEs, a well-known DNA-binding protein used for targeted editing is the zinc finger protein tandem array (ZFA). The DNA recognition domain of ZFA contains 3–6 or more Cys2-His2 zinc fingers. Each zinc finger in a ZFA recognizes a 3-bp DNA sequence via a single α-helix; tandem array assembly allows recognition of a longer sequence in the increments of 3, usually 9–18 bp in length [176]. ZFAs approach the DNA from the major groove [177] and bind with high specificity to DNA sequences [176,178]. Different ZFA modules are used in combination, based on their respective affinities for a particular three-base sequence, to target specific genomic regions [179]. The site-specific DNA demethylation using ZFA-TETs fusion protein is shown in Figure 1A.

Both ZFA and TALE were used as binding platforms for TET1 in site-specific DNA-demethylation studies, and both systems were equally effective in inducing transcription of targeted genes [175,180,181]. ZFAs and TALEs each have strengths and weaknesses regarding opportunities for targeting and specificity of binding; however, ZFAs provide a broader choice of specific arrays for a given target area. Novel algorithms simplify and improve the design process [182,183,184]. In a typical 3-finger array, 9 bp are recognized; however, it is possible to design 6-mers or longer molecules to target 20–30 bp sequences for better specificity. Longer ZFAs and TALEs, however, could still suffer from some non-specific binding, as off-target binding is known to occur both for ZFs [185], and for TALE’s [186,187]. To maximize single gene specificity, a ‘multiple hits’ approach may be used to target multiple fusion molecules to the same promoter in the near vicinity of each other, and in both strands [86], because the action of multiple demethylase molecules is cumulative, and it is unlikely that a combination of a dozen different constructs will bind in close proximity to an unrelated promoter.

In the last decade, the clustered regularly interspaced short palindromic repeat-associated ‘dead’ Cas9 (CRISPR/dCas9) module has received significant attention as a site-specific epigenome editing tool [166,188]. The CRISPR module can be used as a site-specific demethylation tool by combining the specific DNA-binding ability of inactivated ‘dead’ cas9 (dCas9) with demethylase, such as TET1 or TDG. Quite a few targeted demethylation studies have been published using CRISPR-dCas9 systems [143,144,145,146,147,148,149,150,151,152,153,154,155,156,157,158,159,160,161,162,163,164,165,166,167,168,189,190,191,192]. Generally, CRISPR-dCas9-TET1CD fusion proteins are paired with sgRNAs that have programmable 20 nucleotide sequences homologous to the target loci (Figure 1B).

The first report on site-specific demethylation using the CRISPR-dCas9-TET1 system was published in 2016 [192]. In that study, the demethylation and transcription of RANKL, MAGEB2 and MMP2 were promoted by the delivery of dCas9-TET1 constructs to cultured cells (HEK293, SH-SY5Y, HeLe) using plasmid vectors. They also modified the sgRNA by inserting bacteriophage MS2 RNA elements into the conventional sgRNA to improve the efficiency of delivery of the fused proteins to their targeted binding regions. Several other attempts have been made to amplify the response to associated effector modules in the CRSPR system. For example, Taghbalout et al. developed the Casilio-DNA Methylation Editing (ME) platform to amplify the efficacy of effector modules working in the targeted promotor loci [146,193]. Nguyen et al. introduced the Sun Tag linker to the system to recruit multiple copies of VP64, a strong non-epigenetic transcriptional activation domain, at each locus of interest, which improved the efficiency of fused TET1 in inducing demethylation and transcription [190].

The characteristics of the three DNA-binding modalities are summarized in Table 2.

**Figure 2:**
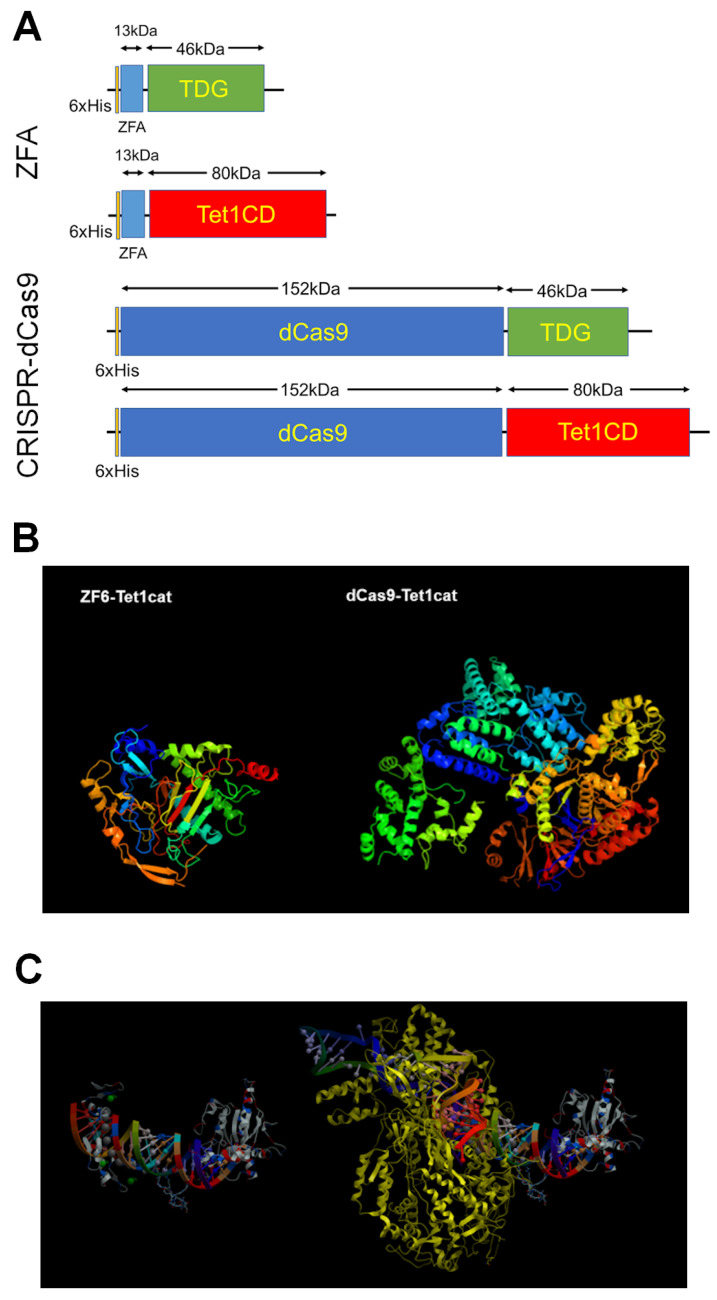
(**A**). Structure and size comparison of ZFA-TDG and ZFA-TET1 constructs. (**B**). Anticipated 3D structural drawings of ZFA-TET1CD and dCAS9-TET1CD constructs. The images were generated with PHYRE2 Protein Fold Recognition Server. (http://www.sbg.bio.ic.ac.uk/phyre2/html/page.cgi?id=index accessed on 20 April 2023). (**C**). Sequence-accurate to-scale 3D rendering of ZFA-TDGCD and dCAS9-TDGCD bound to a target gene promoter. The image was derived from ICM Browser using custom PDB files.

Comparing ZFA with CRISPR, both have their own strengths and weaknesses. ZFA, being a protein, is more stable in vivo than a guide RNA. As shown in Figure 2, ZFAs are much smaller than dCAS9, for which the size and structural complexity represent a significant problem. ZFAs can permeate into cells and further into their nuclei, spontaneously delivering a fused enzymatic payload [194], whereas for dCAS9, additional measures for internalization and translocation are required. At the same time, gRNA DBD promises a better specificity of the binding; re-designing a ZFA-based construct to target a new gene requires re-expression and repurification of the entire fusion protein, whereas, with dCAS9-demethylases, only a replacement of gRNA is required to re-target; finally, the emerging wealth of tools for CRISPR editing creates a favorable landscape for the development of new applications. Nevertheless, the current enthusiasm for CRISPR technology in gene editing cannot be directly translated to vector-free epigenetic editing; the benefits of ZFA over dCAS9 (spontaneous internalization, small size, absence of the need to co-transfect gRNAs and protect them from the aggressive in vivo airway milieu) make it a valuable approach that remains a useful tool for epigenetic editing [195,196].

Finally, some older domains may become re-employed to support the transition of epigenetic editing from virally vectored to protein-only tools. [197].

## 7. In Vivo Delivery

Most of the previous findings on site-targeted DNA demethylation have been based on in vitro experiments. As one of the few exceptions, Lei et al. introduced the CRISPR system into mice by microinjection of the dCas9-DNA methyltransferase MQ1 construct on a lentivirus vector into zygotes [198]. Hanzawa et al. also expressed plasmid-based CRISPR/dCas9 in mouse livers using the hydrodynamic tail vein injection [166]. These studies are highly relevant because they demonstrate that the approach of site-specific DNA demethylation can potentially work in vivo. However, laboratory techniques such as zygotes microinjection and hydrodynamic tail vein injection have problems in clinical application, such as high procedural difficulty and inability to ensure accurate organ-specific delivery. Methods such as microinjection and electroporation are more suitable in vitro [199,200].

Since in vivo knowledge of site-specific DNA demethylation is so limited, an overview of in vivo site-specific demethylation will depend on what is known about targeted nucleases genome editing, which has similarities [198]. Here, we summarized current strategies of three main viral vectors and other non-viral technologies.

### 7.1. Viral Delivery

Viral transduction benefits from the ability of virions to incorporate their genome into the host for replication; thus, viruses are used as vectors to encode proteins and deliver them to target cells.

#### 7.1.1. Adenoviral Vectors (AdVs)

First-generation adenovirus vectors (AdVs) were designed to substitute a transgene for the E1 (3.15 kb) and/or E3 (3.1 kb) regions. [201]. The first-generation adenovirus vectors could transduce a wide variety of target cells, achieved high levels of gene expression and were sufficient for applications where transient high activity was required and an immune response to the vector or transgene was negligible. However, the first generation of adenoviral vectors had to overcome several safety concerns. At high ‘multiplicities of infection’ (MOI), certain host gene products with E1-like activity may be expressed, thereby unnecessarily inducing host cell proliferation [202]. Leakage of adenoviral DNA replication accumulating cytotoxic late gene products has a direct cytotoxic effect on transduced cells and triggers a host cellular immune response [203,204]. To overcome these adverse effects, the second generation of AdVs were developed with extended genome packaging capacity by removing two or more initial genes, such as E2 and E4 [205]. Several clinical trials of genome editing nucleases have been conducted using such AdVs. For systemic delivery of ZFAs, the expression unit coding the ZFAs is inserted into a serotype 5 AdV pseudotyped with serotype 35 fiber (AdV5/35) to repair autologous CD4+ helper T cells from HIV infected patients [206]. Despite improvements compared to the first generation, the problem of immune side effect in vivo has not been completely resolved in the second generation AdVs, and therefore they are not yet optimal vectors for therapeutic approaches [207]. A further evolution of AdVs is the high-capacity AdV (HCAdV), which removes all virus-specific genetic information, leaving only the inverse terminal repeat sequence (ITR) and packing signal. Compared to common AdVs, HCAdVs have the following advantages: they suppress both innate and adaptive immune responses, and their total packaging capacity has been expanded to a maximum of 36 kb, facilitating delivery of large TALE expression cassettes or the CRISPR/dCas system [208].

One drawback of AdVs is that the level of coxsackie-adenovirus receptor (CAR), the receptor for Ad on cells, determines the delivery efficiency [209]. For example, AR expression in cancer cells is said to be negatively correlated with tumor grade, which means that high delivery efficiency may not be achieved when targeting cancer cells in the advanced clinical stage [210]. Therefore, the study of AdVs that could specifically act on cancer cells is taking on a new and important role in anti-tumor research.

#### 7.1.2. Adeno-Associated Viruses Vectors (AAVs)

Adeno-associated viral vectors (AAVs) are delivery vectors derived from icosahedral, non-enveloped viruses of the family Parvoviridae, genus Dependovirus, and are commonly used in genome editing technology because of their ability to be incorporated site-specifically and because of their low immunogenicity. The pathogenicity of AAV has not been confirmed so far. AAVs can deliver their genomes to mitotic and non-mitotic cells and can survive outside the chromosome without delivering their genome to the host cell [211]. A major drawback of the AAV vector system is its relatively small genome size (approximately 4.7 kb), which limits the size of the foreign genes that it can carry. However, with careful design, the sequences for expressing ZFA and TALE expression cassettes can be successfully encapsulated in the AAV vector [207]. Multiple studies on AAV/ZFA-mediated genome editing have shown positive results in experiments with animal models in vivo [212,213,214]. For example, in a study on hemophilia B using a humanized mouse model, the concentration of human factor IX in plasma recovered to 23% of normal on average by administering ZFA-carried AAV to the model mice [215].

Since the size of a typical spCas9 is about 4.2 kb, packaging it using an AAV with a cargo size of about 4.5 kb poses a challenge [215]. To solve this problem, a smaller saCas9 (~3.2 kb) was developed from Staphylococcus aureus species [216]. To date, AAV is established as a delivery vehicle for the CRISPR/Cas system and has been used to target brain cells, as well as skeletal and cardiac muscle cells [217,218].

On another topic, AAV has been reported to be a promising vaccine vector. Taking advantage of this property, attempts are being made to develop a COVID-19 vaccine based on the AAV vector [219,220]. The AAV vaccines reportedly have advantages over first-generation vaccines targeting the same spike protein, such as thermostability, high efficiency, safety and single-dose vaccination. This research demonstrates the potential and benefits of an AAV as a gene transfer vector.

#### 7.1.3. Lentiviral Vectors (LVs)

Lentiviruses are viruses belonging to the retroviridae family and are characterized by the stable insertion of viral genomic information into the host genome using viral RT (reverse transcriptase) and IN (integrase) [221]. Lentivirus-based vectors have attractive advantages as gene transfer vectors, including the following [222]:

(i) sustained gene transfer through stable integration of the vector into the host genome.
(ii) ability to infect both dividing and non-dividing cells.
(iii) broad tissue and cell orientation.
(iv) no expression of viral proteins in the host cells after vector transduction.
(v) ability to deliver complex gene elements.
(vi) a more secure integrated site profile.
(vii) relatively easy vector manipulation and production.

However, multiple patient deaths in early clinical trials have prevented lentiviral use as delivery vectors in humans [223,224]. Therefore, safer integrase-deficient lentiviral vectors (IDLV) have been developed in recent years. For example, Lombardo et al. used IDLV as a delivery vector for ZFA-based genome editing in human stem cells [225]. However, the efficiency of IDLV may depend on the target tissue [224]. For example, IDLV is less efficient at transferring genes into mouse hepatocytes than a conventional lentiviral vector, but it is highly efficient at transferring genes into murine muscle [224,226].

The characteristics of the three vectors mentioned above are summarized in Table 3.

Although viral vectors are by far the most efficient tools for gene transfer, many issues preclude their in vivo and especially clinical use. Therefore, while the transduction/transfection-based approach to embedding epigenetically acting fusion proteins into recipient cells has served a useful purpose in the early stages of epigenetic editing, with experimentation almost exclusively being performed in vitro, it is linked to numerous problems when it comes to translating it into in vivo, and especially into human applications. The viral vector is a foreign invader; thus, the body may mount an immune response [227,228]; a repeat administration or, in case of a pre-existing immunity to the viral vector, even the first administration will be ineffective for an immune subject [227].

Especially with retroviral vectors, genes incorporated into chromosomes pose a risk of oncogenesis due to random gene transfer [229,230,231]. Moreover, insertional mutagenesis, viral persistence, accumulation of proto-oncogenic lesions, immunogenicity, cellular toxicity, risks related to immune surveillance and other problems common to gene therapy will similarly affect epigenetic therapy [232,233,234,235]. Finally, viral transfection can affect transcriptional activity of host genes, which creates the potential for misinterpretation of gene expression data generated from transfected cells [236] and impairs usefulness for experimentation on epigenetic control of transcription, even in vitro but especially in vivo.

### 7.2. Non-Viral Vectors

Non-viral vectors are classified into several subtypes based on their raw materials, sizes or production methods. For example, by raw material they can be classified into lipid-based vectors, polymer-based vectors, etc. Lipid-based vectors are further classified into lipid nanoparticles (LNPs) and liposomes, etc., depending on their manufacturing method and size. Liposomes, polymersomes, lipoplexes, polyplexes and dendrimers, are commonly used as gene delivery vectors and can enclose the mRNA encoding the fusion chimeras [237,238,239,240]. However, the low transfection efficiency and low gene expression rate compared to viral vectors have been obstacles to the clinical application of non-viral vectors. Considering this, various improvements of non-viral vectors have been attempted [241]. One example of these improvements is cationic liposomes that have been validated for their high transfection efficiency and biocompatibility [242]. Positively charged gene particles packaged in cationic lipids or cationic polymers are expected to be attracted to the negative charges of the cell membrane, thus increasing the efficiency of cellular uptake [243]. Studies on non-viral nanoparticle-mediated in vivo delivery are increasing in number, especially for the delivery of relatively small molecules such as RNAi [244,245,246]. However, there are few reports on non-viral vector-mediated in vivo delivery of megamolecules for genome editing. As an example, Han et al. recently reported using nanoparticles to deliver antithrombin-targeted CRISPR/Cas9 to the liver of a hemophiliac model mouse [247]. Several groups have now reported LNP formulations for successful expression in lungs following inhalation [248,249,250,251,252], indicating that the approach may be ready for therapeutic efforts in pulmonary disease. Specifically, in [253], more efficient delivery to fibrotic than healthy lungs was reported, reached over 12% of epithelial cells and 10% endothelial cells in fibrotic lungs following intratracheal delivery.

Applications of programmable nuclease complexes are often hampered by the inability of the complex to reach the target tissue or, if it does reach the target, to pass through the cell membrane and then into the nucleus, and to exert therapeutic activity in vivo. Similar challenges can be expected to occur with site-specific demethylases.

### 7.3. Direct Delivery of Epigenetically Acting Fusion Proteins

Transition from viral vectors to the use of non-viral engineered packaging systems for epigenetically acting constructs could be a step forward in the field. It is important to consider, however, that the very need for genomic integration/transgenesis is linked to some of the side effects and challenges listed above for viral vectors. Lack of control on the site of insertion, problematic control of expression (both the duration of expression and its extent) including inability to stop the action if needed, immunogenicity of the mRNA, design challenges and finally susceptibility to degradation by nucleases are commonly seen with mRNA therapies and vaccines [254]. Thus, development of novel, safe and effective approaches, e.g., based on protein-only ‘biologics’ that act on DNA methylation, is enticing.

Key challenges that may be anticipated here will include ascertaining internalization of such fusion protein constructs into recipient cells and nuclei, and maintaining their intactness in an in vivo environment. Proteins are usually less lipophilic, causing problems in the cell membrane permeation process, but a ZFA protein complex can, surprisingly, penetrate cell membranes due to the positive charge of Cys2-His2 zinc finger domains, and Gaj et al. showed that the direct delivery of ZFA-tethered nuclease protein can disrupt the CCR5 gene in both HEK-293 HDF cells, and human CD4+ T cells [255]. Unlike the naturally cell-penetrating ZFAs, for TALEs and CRISPR/Cas9, conjugation of cell-penetrating peptides (CPPs) is needed to facilitate cell entry [256]. In recent years, various CPPs, such as TAT peptide (YGRKKRRQRRR), have been developed [257].

Another problem with direct delivery, especially for in vivo applications, is protein stability. In the in vivo environment, unprotected proteins will come into direct contact with degrading enzymes. Therefore, it is important to not only expedite delivery, but also to increase the stability of the protein or otherwise protect it, e.g., by encapsulation into vesicles. One method utilized plasma membrane-derived extracellular microvesicles (ARMMs; arrestin domain-containing protein 1-mediated microvesicles) [258,259] for delivery of CRISPR-Cas9/guide RNA complex, which can be employed for epigenetic editing. For ZFAs, strategies to keep them stable have also been proposed, such as modifying the lysine residues necessary for degradation [260]. Finally, the LNP packaging discussed above can also be employed to package not only mRNA, but also the purified epigenetically acting constructs, provided their size allows.

At the moment, non-vectored (ribo)protein-only epigenetic editing is the cutting edge of the field.

## 8. Conclusions

The number of publications on targeted DNA demethylation has increased at an accelerating rate in recent years, reflecting the high level of interest in this field. Most of them are in vitro (Table 4) or ex vivo [106] studies using plasmid or viral vectors as a carrier, but, recently, in vivo experiments have also been reported [143,166]. Although neither of the gene delivery strategies are immediately applicable to clinical research, further expansion of in vivo applications of the targeted DNA demethylation is expected to follow in the future.

Selected targeted DNA demethylation/gene reactivation-related papers are summarized in Table 4.

Targeted DNA demethylation in key regulatory areas of genes allows re-activation of epigenetically silenced transcripts for experimental and therapeutic purposes. This technology can be broadly useful in biomedical research, and its current advances aim to overcome the key challenges of delivery, efficiency and gene-specificity of epigenetic effects.

Summary of benefits:

- Opens the gene to context-dependent, situation-appropriate stimuli
- Potentially heritable (at least mitotically) effects in tissues
- Mechanistic studies in epigenetics (environmental epigenetics, immunotoxicology)
- Vector-free methods will allow studying causality of immunoregulatory genes
- Forms a new class of ‘biologic’ drugs

Summary of challenges:

- Not every gene can likely be targeted
- Gene-specificity/off-target effects
- Non-viral delivery challenges
- Cell-specific delivery

## Figures and Tables

**Table 1:** Reports on disease associated promoter DNA hypermethylation in noncancer diseases.

Category	Disease	Gene	Year	References
nervous	Alzheimer’s disease	HOXB6 ^a^, ANKRD30B ^b^, etc.	2019, 2020	[117,118]
	Parkinson’s disease	Solute Carrier Family 7A11	2020	[119]
	schizophrenia	Solute Carrier Family 6A4	2022	[120]
	borderline personality disorder	BDNF ^c^	2018	[121]
	epilepsy	PABPC1 ^d^, ARGLU1 ^e^, etc.	2021	[122]
	depression	KLK8 ^f^, NR3C1 ^g^	2021	[123,124]
	fragile X syndrome	FMR1 ^h^	2019	[105]
immunological	systemic lupus erythematosus	NCR3 ^i^	2021	[125]
	rheumatoid arthritis	SFRP2 ^j^	2018	[126]
	systemic sclerosis	PARP-1 ^k^	2018	[127]
	Sjogren’s syndrome	IRE1α ^l^, XBP-1 ^m^, GRP78 ^n^	2018	[128]
	Graves’ disease	CRHR1 ^o^, B3GNT2 ^p^	2021	[129]
	type 1 diabetes	Cathepsin H	2021	[130]
	Crohn’s disease	KCNJ15 ^q^	2022	[131]
endocrine/metabolic	osteoporosis	Nrf2 ^r^	2021	[132]
	type 2 diabetes	TXNIP ^s^	2021	[133]
	hyperlipidemia	PPARα ^t^	2022	[134]
cardiovascular	cardiomyopathy	ASB1 ^u^	2018	[135]
renal	IgA nephropathy	Cosmc ^v^	2015	[136]
	diabetic nephropathy	TRIM13 ^w^	2020	[137]
pulmonary	idiopathic pulmonary fibrosis	PPARγ	2022	[138]
	chronic obstructive pulmonary disease	CYP4F11 ^x^, SNRPN ^y^, etc.	2014	[139]
hepatic	hepatitis C	SHP-1 ^z^	2021	[140]

^a^ homeobox B6, ^b^ ankyrin repeat domain 30B, ^c^ brain-derived neurotrophic factor, ^d^ poly(A) binding protein, cytoplasmic 1, ^e^ arginine and glutamate rich 1, ^f^ kallikrein-8, ^g^ Nuclear Receptor Subfamily 3 Group C Member 1, ^h^ FMRP translational regulator 1, ^i^ natural cytotoxicity triggering receptor 3, ^j^ secreted frizzled-related protein 2, ^k^ poly [ADP-ribose] polymerase 1, ^l^ Inositol-requiring enzyme 1, ^m^ X-box binding protein 1, ^n^ glucose regulated protein78, ^o^ corticotropin releasing hormone receptor 1, ^p^ UDP-GlcNAc:betaGal beta-1,3-N-acetylglucosaminyltransferase 2, ^q^ Potassium inwardly rectifying channel, subfamily J, member 15, ^r^ nuclear factor erythroid 2–related factor 2, ^s^ Thioredoxin Interacting Protein, ^t^ peroxisome proliferator-activated receptor alpha. ^u^ ankyrin repeat and SOCS box containing 1, ^v^ core1ß1, 3galactosyl transferase, ^w^ tripartite motif containing 13, ^x^ cytochrome P450, family 4, subfamily F, polypeptide 11, ^y^ Small nuclear ribonucleoprotein-associated protein N, ^z^ Src homology domain 2-containing protein tyrosine phosphatase 1.

**Table 2:** Comparison of three DNA-binding domains for vector-free demethylation.

	ZFA	TALE	CRISPR/dCas9
Components	Zinc finger domain	TALE DNA-binding domain	crRNA/tracrRNA or sgRNA, dCas9
Size	~1 kb + demethylase	~2 kb + demethylase	4.2 kb (dCas9) + 0.1 kb (sgRNA)
Working mechanism	DNA/protein recognition	DNA/protein recognition	DNA/RNA recognition
Length of the target sequence	9–36 bp	30–40 bp per TALE pair	20–22 bp
Target recognition efficiency	High	High	Very High
Off target effects	Low for long arrays	Unknown	Lowest
Multiplexing	Possible	Difficult	Possible
Cloning	Essential	Essential	Not essential
Advantages	Small protein size (<1 kDa) facilitates packaging and purification	High specificity with each module recognizing 1bp; no need to engineer linkage between repeats	Enable multiplexing
	When administered as a naked protein, spontaneously internalize into cells and translocate into nuclei, delivering the fused enzymatic payload		Only a replacement of sgRNA is required to retarget new genes
Limitations	Re-designing the entire protein is required to re-target	Re-designing is required to target new genes	Large protein size (~150 kDa) makes packaging into vectors or nanovesicles difficult
	Cloning methods that require additional linker sequence to fuse modules together add variability	Repetitive sequences may induce undesirable recombination events	When administered as a naked protein, additional measures for cellular internalization and nuclear translocation are required
		Has difficulty binding methylated DNA	sgRNA may be unstable under in vivo and in vitro biological conditions, may require packaging/protection

**Table 3:** The characteristics of the three gene transfer vectors.

	AdVs *	AAVs **	LVs ***
Cell affinity	Inefficient for some types of cells	Dependent on viral serotypes	Broad
Infection into non-dividing cells	+	+	+
Transient/Stable expression	Stable expression by genome integration	Transient, episomal	Transient, episomal
Maximum titer	Very high	High	High
In vivo immunogenicity	High	Very low	Low

* adenoviral vectors. ** adeno-associated viruses vectors. *** lentiviral vectors.

**Table 4:** Selected publications for targeted DNA demethylation/gene reactivation.

DBDs	Enzymes	Targeted Gene	Research Materials	Publication Year	Ref
TALE	TET1	*RHOXF2* ^a^	HeLa cells	2013	[142]
TALE	TET1	*Ascl1* ^b^	Neural stem cells	2017	[261]
ZFA	TDG	*Nos2* ^c^	NIH-3T3 cells	2013	[86]
ZFA	TET2	*C13ORF18* ^d^, *CCNA1* ^e^, *TFPI2* ^f^, *SERPINB5* ^g^	HeLa cells, SiHa cells, CaSki cells, C33a cells	2016	[262]
ZFA	TET1	*FWA* ^h^	Arabidopsis	2016	[263]
CRISPR	TET1	*RANKL* ^i^, *MAGEB2* ^j^, *MMP2* ^k^	HEK293FT cells	2016	[192]
CRISPR	TET1	*H19* ^l^, *RHOXF2*, *CARD9* ^m^, *SH3BP2* ^n^, *CNKSR1* ^o^, *GFAP* ^p^	ESC, cancer cell lines, primary neural precursor cells, mouse fetuses	2016	[143]
CRISPR	TET1	*BRCA1* ^q^	HeLa cells, MCF7 cells	2016	[264]
CRISPR	TET1	*ST6GAL1* ^r^	CHO cells	2018	[165]
CRISPR	TET1	*Oct4* ^s^	NIH-3T3 cells	2022	[164]
CRISPR	TET1	*FOXP3* ^t^	HCC202 cells, HEK293T cells	2022	[154]
CRISPR	TET1	*PPH* ^u^	Arabidopsis	2022	[265]
Rel-homology domain (RHD) of NFκB	TDG	*Nos2*	NIH-3T3 cells	2012	[85]
DNA-binding domain of yeast GAL4	ROS1 5mC DNA glycosylase	Targeted reporter gene	HEK293 cells	2013	[170]
Proteins coded by the genes					

^a^ Rhox homeobox family member 2, ^b^ Achaete-scute homolog 1, ^c^ Nitric oxide synthase 2, ^d^ Chromosome 13 Open Reading Frame 18, ^e^ Cyclin-A1, ^f^ Tissue factor pathway inhibitor 2, ^g^ Maspin, ^h^ homeodomain-containing transcription factor that controls flowering, ^i^ Receptor activator of nuclear factor kappa-Β ligand, ^j^ Melanoma-associated antigen B2, ^k^ Matrix metallopeptidase 2, ^l^ long noncoding RNA, ^m^ Caspase recruitment domain-containing protein 9, ^n^ SH3 domain-binding protein 2, ^o^ Connector enhancer of kinase suppressor of ras 1, ^p^ Glial fibrillary acidic protein, ^q^ Breast cancer type 1 susceptibility protein, ^r^ Beta-galactoside alpha-2,6-sialyltransferase 1, ^s^ octamer-binding transcription factor 4, ^t^ Forkhead box P3, ^u^ Pheophytin pheophorbide hydrolase.

## Data Availability

Not applicable.

## References

[1] Ao C., Gao L., Yu L. (2022). Research Progress in Predicting DNA Methylation Modifications and the Relation with Human Diseases. Curr. Med. Chem..

[2] Salameh Y., Bejaoui Y., El Hajj N. (2020). DNA Methylation Biomarkers in Aging and Age-Related Diseases. Front. Genet..

[3] Ghoshal K., Majumder S., Li Z., Dong X., Jacob S.T. (2000). Suppression of Metallothionein Gene Expression in a Rat Hepatoma Because of Promoter-specific DNA Methylation. J. Biol. Chem..

[4] Jaenisch R., Bird A. (2003). Epigenetic regulation of gene expression: How the genome integrates intrinsic and environmental signals. Nat. Genet..

[5] Meissner A., Mikkelsen T.S., Gu H., Wernig M., Hanna J., Sivachenko A., Zhang X., Bernstein B.E., Nusbaum C., Jaffe D.B. (2008). Genome-scale DNA methylation maps of pluripotent and differentiated cells. Nature.

[6] Jin B., Li Y., Robertson K.D. (2011). DNA methylation: Superior or subordinate in the epigenetic hierarchy?. Genes Cancer.

[7] Smith Z.D., Meissner A. (2013). DNA methylation: Roles in mammalian development. Nat. Rev. Genet..

[8] Onabote O., Hassan H.M., Isovic M., Torchia J. (2022). The Role of Thymine DNA Glycosylase in Transcription, Active DNA Demethylation, and Cancer. Cancers.

[9] Ren W., Gao L., Song J. (2018). Structural Basis of DNMT1 and DNMT3A-Mediated DNA Methylation. Genes.

[10] Guo F., Li X., Liang D., Li T., Zhu P., Guo H., Wu X., Wen L., Gu T.-P., Hu B. (2014). Active and Passive Demethylation of Male and Female Pronuclear DNA in the Mammalian Zygote. Cell Stem Cell.

[11] Suelves M., Carrió E., Núñez-Álvarez Y., Peinado M.A. (2016). DNA methylation dynamics in cellular commitment and differentiation. Briefings Funct. Genom..

[12] Pfeifer G.P., Steigerwald S.D., Grünwald S. (1989). The DNA methylation system in proliferating and differentiated cells. Cell Biochem. Biophys..

[13] Wilks A., Seldran M., Jost J.-P. (1984). An estrogen-dependent demethylation at the 5′ end of the chicken vitellogenin gene is independent of DNA synthesis. Nucleic Acids Res..

[14] Niehrs C. (2009). Active DNA demethylation and DNA repair. Differentiation.

[15] Szyf M., Eliasson L., Mann V., Klein G., Razin A. (1985). Cellular and viral DNA hypomethylation associated with induction of Epstein-Barr virus lytic cycle. Proc. Natl. Acad. Sci. USA.

[16] Mayer W., Niveleau A., Walter J., Fundele R., Haaf T. (2000). Demethylation of the zygotic paternal genome. Nature.

[17] Ou J.-N., Torrisani J., Unterberger A., Provençal N., Shikimi K., Karimi M., Ekström T.J., Szyf M. (2007). Histone deacetylase inhibitor Trichostatin A induces global and gene-specific DNA demethylation in human cancer cell lines. Biochem. Pharmacol..

[18] Gehring M., Huh J.H., Hsieh T.-F., Penterman J., Choi Y., Harada J.J., Goldberg R.B., Fischer R.L. (2006). DEMETER DNA Glycosylase Establishes MEDEA Polycomb Gene Self-Imprinting by Allele-Specific Demethylation. Cell.

[19] Barreto G., Schäfer A., Marhold J., Stach D., Swaminathan S.K., Handa V., Döderlein G., Maltry N., Wu W., Lyko F. (2007). Gadd45a promotes epigenetic gene activation by repair-mediated DNA demethylation. Nature.

[20] Razin A., Szyf M., Kafri T., Roll M., Giloh H., Scarpa S., Carotti D., Cantoni G.L. (1986). Replacement of 5-methylcytosine by cytosine: A possible mechanism for transient DNA demethylation during differentiation. Proc. Natl. Acad. Sci. USA.

[21] Weaver I.C., Cervoni N., Champagne F.A., D’Alessio A.C., Sharma S., Seckl J.R., Dymov S., Szyf M., Meaney M.J. (2004). Epi-genetic programming by maternal behavior. Nat. Neurosci..

[22] Kim S.T., Fields P.E., Flavell R.A. (2007). Demethylation of a specific hypersensitive site in the Th2 locus control region. Proc. Natl. Acad. Sci. USA.

[23] Kangaspeska S., Stride B., Métivier R., Polycarpou-Schwarz M., Ibberson D., Carmouche R.P., Benes V., Gannon F., Reid G. (2008). Transient cyclical methylation of promoter DNA. Nature.

[24] Lu H.-G., Zhan W., Yan L., Qin R.-Y., Yan Y.-P., Yang Z.-J., Liu G.-C., Li G.-Q., Wang H.-F., Li X.-L. (2014). TET1 partially mediates HDAC inhibitor-induced suppression of breast cancer invasion. Mol. Med. Rep..

[25] Bayraktar G., Kreutz M.R. (2018). The Role of Activity-Dependent DNA Demethylation in the Adult Brain and in Neurological Disorders. Front. Mol. Neurosci..

[26] Sleutels F., Barlow D.P. (2002). The origins of genomic imprinting in mammals. Adv. Genet..

[27] Jeziorska D.M., Murray R.J.S., De Gobbi M., Gaentzsch R., Garrick D., Ayyub H., Chen T., Li E., Telenius J., Lynch M. (2017). DNA methylation of intragenic CpG islands depends on their transcriptional activity during differentiation and disease. Proc. Natl. Acad. Sci. USA.

[28] Tang L., Ye H., Hong Q., Wang L., Wang Q., Wang H., Xu L., Bu S., Zhang L., Cheng J. (2014). Elevated CpG island methylation of GCK gene predicts the risk of type 2 diabetes in Chinese males. Gene.

[29] de la Rocha C., Zaina S., Lund G. (2020). Is Any Cardiovascular Disease-Specific DNA Methylation Biomarker Within Reach?. Curr. Atheroscler. Rep..

[30] Kitamoto T., Kitamoto A., Ogawa Y., Honda Y., Imajo K., Saito S., Yoneda M., Nakamura T., Nakajima A., Hotta K. (2015). Targeted-bisulfite sequence analysis of the methylation of CpG islands in genes encoding PNPLA3, SAMM50, and PARVB of patients with non-alcoholic fatty liver disease. J. Hepatol..

[31] Feinberg A.P., Vogelstein B. (1983). Hypomethylation distinguishes genes of some human cancers from their normal counterparts. Nature.

[32] Xue Y., Yang Y., Tian H., Quan H., Liu S., Zhang L., Yang L., Zhu H., Wu H., Gao Y.Q. (2021). Computational characterization of domain-segregated 3D chromatin structure and segmented DNA methylation status in carcinogenesis. Mol. Oncol..

[33] Chen Y.-T., Lin W.-D., Liao W.-L., Lin Y.-J., Chang J.-G., Tsai F.-J. (2015). PTPRD silencing by DNA hypermethylation decreases insulin receptor signaling and leads to type 2 diabetes. Oncotarget.

[34] Tsuboi Y., Yamada H., Munetsuna E., Fujii R., Yamazaki M., Ando Y., Mizuno G., Ishikawa H., Ohashi K., Hashimoto S. (2021). Global DNA hypermethylation in peripheral blood mononuclear cells and cardiovascular disease risk: A population-based propensity score-matched cohort study. J. Epidemiol. Community Health.

[35] Smyth L.J., McKay G.J., Maxwell A.P., McKnight A.J. (2013). DNA hypermethylation and DNA hypomethylation is present at different loci in chronic kidney disease. Epigenetics.

[36] Dinardo A.R., Rajapakshe K., Nishiguchi T., Grimm S.L., Mtetwa G., Dlamini Q., Kahari J., Mahapatra S., Kay A.W., Maphalala G. (2020). DNA hypermethylation during tuberculosis dampens host immune responsiveness. J. Clin. Investig..

[37] Chen H.-C., Chen Y.-Z., Wang C.-H., Lin F.-J. (2019). The nonalcoholic fatty liver disease-like phenotype and lowered serum VLDL are associated with decreased expression and DNA hypermethylation of hepatic ApoB in male offspring of ApoE deficient mothers fed a with Western diet. J. Nutr. Biochem..

[38] Xiang T., Li L., Yin X., Yuan C., Tan C., Su X., Xiong L., Putti T.C., Oberst M., Kelly K. (2012). The Ubiquitin Peptidase UCHL1 Induces G0/G1 Cell Cycle Arrest and Apoptosis Through Stabilizing p53 and Is Frequently Silenced in Breast Cancer. PLoS ONE.

[39] Saavedra K., Valbuena J., Olivares W., Marchant M.J., Rodríguez A., Torres-Estay V., Carrasco-Aviño G., Guzmán L., Aguayo F., Roa J.C. (2015). Loss of Expression of Reprimo, a p53-induced Cell Cycle Arrest Gene, Correlates with Invasive Stage of Tumor Progression and p73 Expression in Gastric Cancer. PLoS ONE.

[40] Xu G., Fan L., Zhao S., OuYang C. (2021). Neuronal pentraxin II (NPTX2) hypermethylation promotes cell proliferation but inhibits cell cycle arrest and apoptosis in gastric cancer cells by suppressing the p53 signaling pathway. Bioengineered.

[41] Keller J.A., Erson-Bensan A.E., Petty E.M. (2010). Connections between CHFR, the cell cycle, and chemosensitivity: Are they critical in cancer?. Cancer Biol. Ther..

[42] Johnson B.E. (2015). Emerging gene mutation targets in lung cancer. Clin. Adv. Hematol. Oncol..

[43] Takeshita M., Koga T., Takayama K., Yano T., Maehara Y., Nakanishi Y., Sueishi K. (2010). Alternative efficacy-predicting markers for paclitaxel instead of CHFR in non-small-cell lung cancer. Cancer Biol. Ther..

[44] Toyota M., Sasaki Y., Satoh A., Ogi K., Kikuchi T., Suzuki H., Mita H., Tanaka N., Itoh F., Issa J.-P.J. (2003). Epigenetic inactivation of CHFR in human tumors. Proc. Natl. Acad. Sci. USA.

[45] Scolnick D.M., Halazonetis T.D. (2000). Chfr defines a mitotic stress checkpoint that delays entry into metaphase. Nature.

[46] Cheng Z.-D., Hu S.-L., Sun Y.-B., Xu W.-P., Shen G., Kong X.-Y. (2010). Promoter methylation of CHFR gene in gastric carcinoma tissues detected using two methods. Chin. J. Cancer.

[47] Castiel A., Danieli M.M., David A., Moshkovitz S., Aplan P.D., Kirsch I.R., Brandeis M., Krämer A., Izraeli S. (2011). The Stil protein regulates centrosome integrity and mitosis through suppression of Chfr. J. Cell Sci..

[48] Erson A.E., Petty E.M. (2003). CHFR-associated early G2/M checkpoint defects in breast cancer cells. Mol. Carcinog..

[49] Yanokura M., Banno K., Kawaguchi M., Hirao N., Hirasawa A., Susumu N., Tsukazaki K., Aoki D. (2007). Relationship of aberrant DNA hypermethylation of CHFR with sensitivity to taxanes in endometrial cancer. Oncol. Rep..

[50] Helling B.A., Gerber N.A., Kadiyala V., Sasse S.K., Pedersen B.S., Sparks L., Nakano Y., Okamoto T., Evans C.M., Yang I.V. (2017). Regulation of MUC5B expression in idiopathic pulmonary fibrosis. Am. J. Respir. Cell Mol. Biol..

[51] Wang H., Maurano M.T., Qu H., Varley K.E., Gertz J., Pauli F., Lee K., Canfield T., Weaver M., Sandstrom R. (2012). Widespread plasticity in CTCF occupancy linked to DNA methylation. Genome Res..

[52] Murayama A., Sakura K., Nakama M., Yasuzawa-Tanaka K., Fujita E., Tateishi Y., Wang Y., Ushijima T., Baba T., Shibuya K. (2006). A specific CpG site demethylation in the human interleukin 2 gene promoter is an epigenetic memory. EMBO J..

[53] Green B.B., Marsit C.J. (2015). Select Prenatal Environmental Exposures and Subsequent Alterations of Gene-Specific and Repetitive Element DNA Methylation in Fetal Tissues. Curr. Environ. Health Rep..

[54] Baccarelli A., Bollati V. (2009). Epigenetics and environmental chemicals. Curr. Opin. Pediatr..

[55] Mund C., Hackanson B., Stresemann C., Lübbert M., Lyko F. (2005). Characterization of DNA Demethylation Effects Induced by 5-Aza-2′-Deoxycytidine in Patients with Myelodysplastic Syndrome. Cancer Res..

[56] Patra S.K., Bettuzzi S. (2009). Epigenetic DNA-(cytosine-5-carbon) modifications: 5-aza-2′-deoxycytidine and DNA-demethylation. Biochemistry.

[57] Singh K.P., Treas J., Tyagi T., Gao W. (2012). DNA demethylation by 5-aza-2-deoxycytidine treatment abrogates 17 beta-estradiol-induced cell growth and restores expression of DNA repair genes in human breast cancer cells. Cancer Lett..

[58] Gnyszka A., Jastrzebski Z., Flis S. (2013). DNA methyltransferase inhibitors and their emerging role in epigenetic therapy of cancer. Anticancer Res..

[59] Stewart D.J., Issa J.-P., Kurzrock R., Nunez M.I., Jelinek J., Hong D., Oki Y., Guo Z., Gupta S., Wistuba I.I. (2009). Decitabine Effect on Tumor Global DNA Methylation and Other Parameters in a Phase I Trial in Refractory Solid Tumors and Lymphomas. Clin. Cancer Res..

[60] Schuermann D., Weber A.R., Schär P. (2016). Active DNA demethylation by DNA repair: Facts and uncertainties. DNA Repair..

[61] De Groote M.L., Verschure P.J., Rots M.G. (2012). Epigenetic Editing: Targeted rewriting of epigenetic marks to modulate expression of selected target genes. Nucleic Acids Res..

[62] Gjerset R.A., Martin D.W. (1982). Presence of a DNA demethylating activity in the nucleus of murine erythroleukemic cells. J. Biol. Chem..

[63] Kavoosi S., Sudhamalla B., Dey D., Shriver K., Arora S., Sappa S., Islam K. (2019). Site- and degree-specific C–H oxidation on 5-methylcytosine homologues for probing active DNA demethylation. Chem. Sci..

[64] Wu H., Zhang Y. (2011). Mechanisms and functions of Tet protein-mediated 5-methylcytosine oxidation. Genes Dev..

[65] Ito S., D’alessio A.C., Taranova O.V., Hong K., Sowers L.C., Zhang Y. (2010). Role of Tet proteins in 5mC to 5hmC conversion, ES-cell self-renewal and inner cell mass specification. Nature.

[66] Tahiliani M., Koh K.P., Shen Y., Pastor W.A., Bandukwala H., Brudno Y., Agarwal S., Iyer L.M., Liu D.R., Aravind L. (2009). Conversion of 5-Methylcytosine to 5-Hydroxymethylcytosine in Mammalian DNA by MLL Partner TET1. Science.

[67] DeNizio J.E., Dow B.J., Serrano J.C., Ghanty U., Drohat A.C., Kohli R.M. (2021). TET-TDG Active DNA Demethylation at CpG and Non-CpG Sites. J. Mol. Biol..

[68] Lorsbach R.B., Moore J., Mathew S., Raimondi S.C., Mukatira S.T., Downing J.R. (2003). TET1, a member of a novel protein family, is fused to MLL in acute myeloid leukemia containing the t(10;11)(q22;q23). Leukemia.

[69] Guo J.U., Su Y., Zhong C., Ming G.-L., Song H. (2011). Hydroxylation of 5-Methylcytosine by TET1 Promotes Active DNA Demethylation in the Adult Brain. Cell.

[70] Seiler C.L., Fernandez J., Koerperich Z., Andersen M.P., Kotandeniya D., Nguyen M.E., Sham Y., Tretyakova N.Y. (2018). Maintenance DNA Methyltransferase Activity in the Presence of Oxidized Forms of 5-Methylcytosine: Structural Basis for Ten Eleven Translocation-Mediated DNA Demethylation. Biochemistry.

[71] Ross S.E., Bogdanovic O. (2019). TET enzymes, DNA demethylation and pluripotency. Biochem. Soc. Trans..

[72] Delatte B., Fuks F. (2013). TET proteins: On the frenetic hunt for new cytosine modifications. Briefings Funct. Genom..

[73] Seethy A., Pethusamy K., Chattopadhyay I., Sah R., Chopra A., Dhar R., Karmakar S. (2021). TETology: Epigenetic Mastermind in Action. Appl. Biochem. Biotechnol..

[74] Akahori H., Guindon S., Yoshizaki S., Muto Y. (2015). Molecular Evolution of the TET Gene Family in Mammals. Int. J. Mol. Sci..

[75] Li D., Guo B., Wu H., Tan L., Lu Q. (2015). TET Family of Dioxygenases: Crucial Roles and Underlying Mechanisms. Cytogenet. Genome Res..

[76] Fromme J., Verdine G.L. (2004). Base Excision Repair. Cold Spring Harb. Perspect. Biol..

[77] Mol C.D., Hosfield D.J., A Tainer J. (2000). Abasic site recognition by two apurinic/apyrimidinic endonuclease families in DNA base excision repair: The 3′ ends justify the means. Mutat. Res. Repair.

[78] Ronemus M.J., Galbiati M., Ticknor C., Chen J., Dellaporta S.L. (1996). Demethylation-Induced Developmental Pleiotropy in *Arabidopsis*. Science.

[79] Zhu B., Zheng Y., Hess D., Angliker H., Schwarz S., Siegmann M., Thiry S., Jost J.-P. (2000). 5-Methylcytosine-DNA glycosylase activity is present in a cloned G/T mismatch DNA glycosylase associated with the chicken embryo DNA demethylation complex. Proc. Natl. Acad. Sci. USA.

[80] Hashimoto H., Hong S., Bhagwat A.S., Zhang X., Cheng X. (2012). Excision of 5-hydroxymethyluracil and 5-carboxylcytosine by the thymine DNA glycosylase domain: Its structural basis and implications for active DNA demethylation. Nucleic Acids Res..

[81] Zhang L., Lu X., Lu J., Liang H., Dai Q., Xu G.-L., Luo C., Jiang H., He C. (2012). Thymine DNA glycosylase specifically recognizes 5-carboxylcytosine-modified DNA. Nat. Chem. Biol..

[82] Hashimoto H., Zhang X., Cheng X. (2013). Selective Excision of 5-Carboxylcytosine by a Thymine DNA Glycosylase Mutant. J. Mol. Biol..

[83] Bennett M.T., Rodgers M.T., Hebert A.S., Ruslander L.E., Eisele L., Drohat A.C. (2006). Specificity of Human Thymine DNA Glycosylase Depends on *N*-Glycosidic Bond Stability. J. Am. Chem. Soc..

[84] Onodera A., González-Avalos E., Lio C.-W.J., Georges R.O., Bellacosa A., Nakayama T., Rao A. (2021). Roles of TET and TDG in DNA demethylation in proliferating and non-proliferating immune cells. Genome Biol..

[85] Gregory D.J., Mikhaylova L., Fedulov A.V. (2012). Selective DNA demethylation by fusion of TDG with a sequence-specific DNA-binding domain. Epigenetics.

[86] Gregory D.J., Zhang Y., Kobzik L., Fedulov A.V. (2013). Specific transcriptional enhancement of inducible nitric oxide synthase by targeted promoter demethylation. Epigenetics.

[87] Hassan H.M., Kolendowski B., Isovic M., Bose K., Dranse H.J., Sampaio A.V., Underhill T.M., Torchia J. (2017). Regulation of Active DNA Demethylation through RAR-Mediated Recruitment of a TET/TDG Complex. Cell Rep..

[88] Weber A.R., Krawczyk C., Robertson A.B., Kuśnierczyk A., Vågbø C.B., Schuermann D., Klungland A., Schär P. (2016). Biochemical reconstitution of TET1–TDG–BER-dependent active DNA demethylation reveals a highly coordinated mechanism. Nat. Commun..

[89] Cortellino S., Xu J., Sannai M., Moore R., Caretti E., Cigliano A., Le Coz M., Devarajan K., Wessels A., Soprano D. (2011). Thymine DNA Glycosylase Is Essential for Active DNA Demethylation by Linked Deamination-Base Excision Repair. Cell.

[90] Niehrs C., Schäfer A. (2012). Active DNA demethylation by Gadd45 and DNA repair. Trends Cell Biol..

[91] Rai K., Huggins I.J., James S.R., Karpf A.R., Jones D.A., Cairns B.R. (2008). DNA Demethylation in Zebrafish Involves the Coupling of a Deaminase, a Glycosylase, and Gadd45. Cell.

[92] Jin S.-G., Guo C., Pfeifer G.P. (2008). GADD45A Does Not Promote DNA Demethylation. PLOS Genet..

[93] Wong E., Yang K., Kuraguchi M., Werling U., Avdievich E., Fan K., Fazzari M., Jin B., Brown A.M.C., Lipkin M. (2002). Mbd4 inactivation increases C→T transition mutations and promotes gastrointestinal tumor formation. Proc. Natl. Acad. Sci. USA.

[94] Hackett J.A., Sengupta R., Zylicz J.J., Murakami K., Lee C., Down T.A., Surani M.A. (2013). Germline DNA Demethylation Dynamics and Imprint Erasure Through 5-Hydroxymethylcytosine. Science.

[95] Yamaguchi S., Hong K., Liu R., Inoue A., Shen L., Zhang K., Zhang Y. (2013). Dynamics of 5-methylcytosine and 5-hydroxymethylcytosine during germ cell reprogramming. Cell Res..

[96] Chen C.-C., Wang K.-Y., Shen C.-K.J. (2013). DNA 5-Methylcytosine Demethylation Activities of the Mammalian DNA Methyltransferases. J. Biol. Chem..

[97] Waheed S.O., Ramanan R., Chaturvedi S.S., Lehnert N., Schofield C.J., Christov C.Z., Karabencheva-Christova T.G. (2020). Role of Structural Dynamics in Selectivity and Mechanism of Non-heme Fe(II) and 2-Oxoglutarate-Dependent Oxygenases Involved in DNA Repair. ACS Central Sci..

[98] Bian K., Lenz S.A.P., Tang Q., Chen F., Qi R., Jost M., Drennan C.L., Essigmann J.M., Wetmore S.D., Li D. (2019). DNA repair enzymes ALKBH2, ALKBH3, and AlkB oxidize 5-methylcytosine to 5-hydroxymethylcytosine, 5-formylcytosine and 5-carboxylcytosine in vitro. Nucleic Acids Res..

[99] Liutkevičiūtė Z., Kriukienė E., Ličytė J., Rudytė M., Urbanavičiūtė G., Klimašauskas S. (2014). Direct Decarboxylation of 5-Carboxylcytosine by DNA C5- Methyltransferases. J. Am. Chem. Soc..

[100] Tsai W.C., Strieter R.M., A Zisman D., Wilkowski J.M., A Bucknell K., Chen G.H., Standiford T.J. (1997). Nitric oxide is required for effective innate immunity against Klebsiella pneumoniae. Infect. Immun..

[101] Richardson A.R., Libby S.J., Fang F.C. (2008). A Nitric Oxide–Inducible Lactate Dehydrogenase Enables *Staphylococcus aureus* to Resist Innate Immunity. Science.

[102] Richardson A.R., Payne E.C., Younger N., Karlinsey J.E., Thomas V.C., Becker L.A., Navarre W.W., Castor M.E., Libby S.J., Fang F.C. (2011). Multiple Targets of Nitric Oxide in the Tricarboxylic Acid Cycle of Salmonella enterica Serovar Typhimurium. Cell Host Microbe.

[103] Sun A., Li Z. (2013). Regulatory role of nitric oxide in lipopolysaccharides-triggered plant innate immunity. Plant Signal. Behav..

[104] Förstermann U., Li H. (2011). Therapeutic effect of enhancing endothelial nitric oxide synthase (eNOS) expression and preventing eNOS uncoupling. Br. J. Pharmacol..

[105] Kraan C.M., E Godler D., Amor D.J. (2018). Epigenetics of fragile X syndrome and fragile X-related disorders. Dev. Med. Child Neurol..

[106] Liu X.S., Wu H., Krzisch M., Wu X., Graef J., Muffat J., Hnisz D., Li C.H., Yuan B., Xu C. (2018). Rescue of Fragile X Syndrome Neurons by DNA Methylation Editing of the FMR1 Gene. Cell.

[107] Weisenberger D.J., Liang G., Lenz H.-J. (2017). DNA methylation aberrancies delineate clinically distinct subsets of colorectal cancer and provide novel targets for epigenetic therapies. Oncogene.

[108] Mann J., Oakley F., Akiboye F., Elsharkawy A., Thorne A.W., A Mann D. (2006). Regulation of myofibroblast transdifferentiation by DNA methylation and MeCP2: Implications for wound healing and fibrogenesis. Cell Death Differ..

[109] Mathison M., Sanagasetti D., Singh V.P., Pugazenthi A., Pinnamaneni J.P., Ryan C.T., Yang J., Rosengart T.K. (2021). Fibroblast transition to an endothelial “trans” state improves cell reprogramming efficiency. Sci. Rep..

[110] Li X. (2019). Epigenetics and cell cycle regulation in cystogenesis. Cell Signal..

[111] Dong Y., Zhao H., Li H., Li X., Yang S. (2014). DNA methylation as an early diagnostic marker of cancer (Review). Biomed. Rep..

[112] Li D., Zhang L., Liu Y., Sun H., Onwuka J.U., Zhao Z., Tian W., Xu J., Zhao Y., Xu H. (2019). Specific DNA methylation markers in the diagnosis and prognosis of esophageal cancer. Aging.

[113] Tserpeli V., Stergiopoulou D., Londra D., Giannopoulou L., Buderath P., Balgkouranidou I., Xenidis N., Grech C., Obermayr E., Zeillinger R. (2021). Prognostic Significance of *SLFN11* Methylation in Plasma Cell-Free DNA in Advanced High-Grade Serous Ovarian Cancer. Cancers.

[114] Pangeni R.P., Zhang Z., Alvarez A.A., Wan X., Sastry N., Lu S., Shi T., Huang T., Lei C.X., James C.D. (2018). Genome-wide methylomic and transcriptomic analyses identify subtype-specific epigenetic signatures commonly dysregulated in glioma stem cells and glioblastoma. Epigenetics.

[115] Shi Y., Li M., Song C., Xu Q., Huo R., Shen L., Xing Q., Cui D., Li W., Zhao J. (2017). Combined study of genetic and epigenetic biomarker risperidone treatment efficacy in Chinese Han schizophrenia patients. Transl. Psychiatry.

[116] Falahi F., Sgro A., Blancafort P. (2015). Epigenome Engineering in Cancer: Fairytale or a Realistic Path to the Clinic?. Front. Oncol..

[117] Roubroeks J.A., Smith A.R., Smith R.G., Pishva E., Ibrahim Z., Sattlecker M., Hannon E.J., Kłoszewska I., Mecocci P., Soininen H. (2020). An epigenome-wide association study of Alzheimer’s disease blood highlights robust DNA hypermethylation in the HOXB6 gene. Neurobiol. Aging.

[118] Semick S.A., Bharadwaj R.A., Collado-Torres L., Tao R., Shin J.H., Deep-Soboslay A., Weiss J.R., Weinberger D.R., Hyde T.M., Kleinman J.E. (2019). Integrated DNA methylation and gene expression profiling across multiple brain regions implicate novel genes in Alzheimer’s disease. Acta Neuropathol..

[119] Vallerga C.L., Zhang F., Fowdar J., McRae A.F., Qi T., Nabais M.F., Zhang Q., Kassam I., Henders A.K., Wallace L. (2020). Analysis of DNA methylation associates the cystine–glutamate antiporter SLC7A11 with risk of Parkinson’s disease. Nat. Commun..

[120] Liu L., Hu Y., Lu Y., Hu L., Gao C., Nie S. (2021). Sex-dependent DNA hypermethylation of SLC6A4 in patients with schizophrenia. Neurosci. Lett..

[121] Thomas M., Knoblich N., Wallisch A., Glowacz K., Becker-Sadzio J., Gundel F., Brückmann C., Nieratschker V. (2018). Increased BDNF methylation in saliva, but not blood, of patients with borderline personality disorder. Clin. Epigenetics.

[122] Zhang W., Wang H., Liu B., Jiang M., Gu Y., Yan S., Han X., Hou A.Y., Tang C., Jiang Z. (2021). Differential DNA Methylation Profiles in Patients with Temporal Lobe Epilepsy and Hippocampal Sclerosis ILAE Type I. J. Mol. Neurosci..

[123] Dereix A.E., Ledyard R., Redhunt A.M., Bloomquist T.R., Brennan K.J., A Baccarelli A., Hacker M.R., Burris H.H. (2021). Maternal anxiety and depression in pregnancy and DNA methylation of the *NR3C1* glucocorticoid receptor gene. Epigenomics.

[124] Starnawska A., Bukowski L., Chernomorchenko A., Elfving B., Müller H.K., Oord E.V.D., Aberg K., Guintivano J., Grove J., Mors O. (2021). DNA methylation of the KLK8 gene in depression symptomatology. Clin. Epigenetics.

[125] Gao B. (2021). Identification of Feature Autophagy-Related Genes and DNA Methylation Profiles in Systemic Lupus Erythematosus Patients. Experiment.

[126] Miao C., Chang J., Dou J., Xiong Y., Zhou G. (2018). DNA hypermethylation of SFRP2 influences the pathology of rheumatoid arthritis through the canonical Wnt signaling in model rats. Autoimmunity.

[127] Zhang Y., Pötter S., Chen C.-W., Liang R., Gelse K., Ludolph I., Horch R.E., Distler O., Schett G., Distler J.H.W. (2018). Poly(ADP-ribose) polymerase-1 regulates fibroblast activation in systemic sclerosis. Ann. Rheum. Dis..

[128] Sepúlveda D., Barrera M.-J., Castro I., Aguilera S., Carvajal P., Lagos C., González S., Albornoz N., Bahamondes V., Quest A.F.G. (2018). Impaired IRE1α/XBP-1 pathway associated to DNA methylation might contribute to salivary gland dysfunction in Sjögren’s syndrome patients. Rheumatology.

[129] Cai T., Qin Q., Song R., Zhao J., Wang G., Zhang J. (2021). Identifying and Validating Differentially Methylated Regions in Newly Diagnosed Patients with Graves’ Disease. DNA Cell Biol..

[130] Ye J., Stefan-Lifshitz M., Tomer Y. (2021). Genetic and environmental factors regulate the type 1 diabetes gene CTSH via differential DNA methylation. J. Biol. Chem..

[131] Wu H., Liu H., Liu H., Chen Y., Liu T., Shen X., Liu L. (2022). Genome-wide DNA methylation profiling in differentiating Crohn’s disease from intestinal tuberculosis. Genes Genom..

[132] Chen X., Zhu X., Wei A., Chen F., Gao Q., Lu K., Jiang Q., Cao W. (2021). Nrf2 epigenetic derepression induced by running exercise protects against osteoporosis. Bone Res..

[133] Wang Z., Peng H., Gao W., Cao W., Lv J., Yu C., Huang T., Sun D., Wang B., Liao C. (2021). Blood DNA methylation markers associated with type 2 diabetes, fasting glucose, and HbA1c levels: An epigenome-wide association study in 316 adult twin pairs. Genomics.

[134] Ando Y., Yamada H., Munetsuna E., Yamazaki M., Kageyama I., Teshigawara A., Nouchi Y., Fujii R., Mizuno G., Sadamoto N. (2022). Maternal high-fructose corn syrup consumption causes insulin resistance and hyperlipidemia in offspring via DNA methylation of the Pparα promoter region. J. Nutr. Biochem..

[135] Ortega A., Tarazón E., Gil-Cayuela C., Martínez-Dolz L., Lago F., González-Juanatey J.R., Sandoval J., Portolés M., Roselló-Lletí E., Rivera M. (2018). *ASB1* differential methylation in ischaemic cardiomyopathy: Relationship with left ventricular performance in end-stage heart failure patients. ESC Heart Fail..

[136] Sun Q., Zhang J., Zhou N., Liu X., Shen Y. (2015). DNA Methylation in Cosmc Promoter Region and Aberrantly Glycosylated IgA1 Associated with Pediatric IgA Nephropathy. PLoS ONE.

[137] Li Y., Ren D., Shen Y., Zheng X., Xu G. (2020). Altered DNA methylation of TRIM13 in diabetic nephropathy suppresses mesangial collagen synthesis by promoting ubiquitination of CHOP. Ebiomedicine.

[138] Wei A., Gao Q., Chen F., Zhu X., Chen X., Zhang L., Su X., Dai J., Shi Y., Cao W. (2022). Inhibition of DNA methylation de-represses peroxisome proliferator-activated receptor-γ and attenuates pulmonary fibrosis. Br. J. Pharmacol..

[139] Vucic E.A., Chari R., Thu K.L., Wilson I.M., Cotton A.M., Kennett J.Y., Zhang M., Lonergan K.M., Steiling K., Brown C. (2014). DNA Methylation Is Globally Disrupted and Associated with Expression Changes in Chronic Obstructive Pulmonary Disease Small Airways. Am. J. Respir. Cell Mol. Biol..

[140] Devi P., Ota S., Punga T., Bergqvist A. (2021). Hepatitis C Virus Core Protein Down-Regulates Expression of Src-Homology 2 Domain Containing Protein Tyrosine Phosphatase by Modulating Promoter DNA Methylation. Viruses.

[141] Chen H., Kazemier H.G., De Groote M.L., Ruiters M.H.J., Xu G.-L., Rots M.G. (2013). Induced DNA demethylation by targeting Ten-Eleven Translocation 2 to the human ICAM-1 promoter. Nucleic Acids Res..

[142] Maeder M.L., Angstman J.F., Richardson M.E., Linder S.J., Cascio V.M., Tsai S.Q., Ho Q.H., Sander J.D., Reyon D., Bernstein B.E. (2013). Targeted DNA demethylation and activation of endogenous genes using programmable TALE-TET1 fusion proteins. Nat. Biotechnol..

[143] Morita S., Noguchi H., Horii T., Nakabayashi K., Kimura M., Okamura K., Sakai A., Nakashima H., Hata K.N.K., Nakashima K. (2016). Targeted DNA demethylation in vivo using dCas9–peptide repeat and scFv–TET1 catalytic domain fusions. Nat. Biotechnol..

[144] Morita S., Horii T., Hatada I. (2018). Editing of DNA Methylation Using dCas9-Peptide Repeat and scFv-TET1 Catalytic Domain Fusions. Epigenome Ed. Methods Protoc..

[145] Morita S., Horii T., Kimura M., Hatada I. (2020). Synergistic Upregulation of Target Genes by TET1 and VP64 in the dCas9–SunTag Platform. Int. J. Mol. Sci..

[146] Taghbalout A., Du M., Jillette N., Rosikiewicz W., Rath A., Heinen C.D., Li S., Cheng A.W. (2019). Enhanced CRISPR-based DNA demethylation by Casilio-ME-mediated RNA-guided coupling of methylcytosine oxidation and DNA repair pathways. Nat. Commun..

[147] Chan W.F., Coughlan H.D., Chen Y., Keenan C.R., Smyth G.K., Perkins A.C., Johanson T.M., Allan R.S. (2022). Activation of stably silenced genes by recruitment of a synthetic de-methylating module. Nat. Commun..

[148] Wilk C., Effenberg L., Abberger H., Steenpass L., Hansen W., Zeschnigk M., Kirschning C., Buer J., Kehrmann J. (2021). CRISPR/Cas9-mediated demethylation of FOXP3-TSDR toward Treg-characteristic programming of Jurkat T cells. Cell Immunol..

[149] Okada M., Kanamori M., Someya K., Nakatsukasa H., Yoshimura A. (2017). Stabilization of Foxp3 expression by CRISPR-dCas9-based epigenome editing in mouse primary T cells. Epigenetics Chromatin.

[150] Xu C., Jiang S., Ma X., Jiang Z., Pan Y., Li X., Zhang L., Zhou H., Chen S., Xing X. (2022). CRISPR-based DNA methylation editing of NNT rescues the cisplatin resistance of lung cancer cells by reducing autophagy. Arch. Toxicol..

[151] Zhang W., Cao H., Yang J., Zhao J., Liang Z., Kang X., Wang R. (2022). The identification and validation of EphA7 hypermethylation, a novel biomarker, in cervical cancer. BMC Cancer.

[152] Lindström A.K., Ekman K., Stendahl U., Tot T., Henriksson R., Hedman H., Hellberg D. (2008). LRIG1 and squamous epithelial uterine cervical cancer: Correlation to prognosis, other tumor markers, sex steroid hormones, and smoking. Int. J. Gynecol. Cancer.

[153] Umeh-Garcia M., O’geen H., Simion C., Gephart M.H., Segal D.J., Sweeney C.A. (2022). Aberrant promoter methylation contributes to LRIG1 silencing in basal/triple-negative breast cancer. Br. J. Cancer.

[154] Cui X., Zhang C., Xu Z., Wang S., Li X., Stringer-Reasor E., Bae S., Zeng L., Zhao D., Liu R. (2022). Dual CRISPR interference and activation for targeted reactivation of X-linked endogenous FOXP3 in human breast cancer cells. Mol. Cancer.

[155] Hatakeyama S. (2017). TRIM Family Proteins: Roles in Autophagy, Immunity, and Carcinogenesis. Trends Biochem. Sci..

[156] Gan Y., Cao C., Li A., Song H., Kuang G., Ma B., Zhang Q. (2021). Silencing of the TRIM58 Gene by Aberrant Promoter Methylation is Associated with a Poor Patient Outcome and Promotes Cell Proliferation and Migration in Clear Cell Renal Cell Carcinoma. Front. Mol. Biosci..

[157] Iżykowska K., Rassek K., Żurawek M., Nowicka K., Paczkowska J., Ziółkowska-Suchanek I., Podralska M., Dzikiewicz-Krawczyk A., Joks M., Olek-Hrab K. (2020). Hypomethylation of the promoter region drives ectopic expression of *TMEM244* in Sézary cells. J. Cell. Mol. Med..

[158] Josipović G., Tadić V., Klasić M., Zanki V., Bečeheli I., Chung F., Ghantous A., Keser T., Madunić J., Bošković M. (2019). Antagonistic and synergistic epigenetic modulation using orthologous CRISPR/dCas9-based modular system. Nucleic Acids Res..

[159] Le Berre G., Hossard V., Riou J.-F., Guieysse-Peugeot A.-L. (2019). Repression of TERRA Expression by Subtelomeric DNA Methylation Is Dependent on NRF1 Binding. Int. J. Mol. Sci..

[160] Fang S., Cui D., Hong T., Guo L., Lee Y.-T., Lee M., Isgandarova S., Martinez-Moczygemba M., Zhou Y., Li J. (2022). Ten-Eleven Translocation Ablation Impairs Cardiac Differentiation of Mouse Embryonic Stem Cells. Stem Cells.

[161] Halmai J.A.N.M., Deng P., E Gonzalez C., Coggins N.B., Cameron D., Carter J.L., Buchanan F.K.B., Waldo J.J., Lock S.R., Anderson J.D. (2020). Artificial escape from XCI by DNA methylation editing of the CDKL5 gene. Nucleic Acids Res..

[162] Kyle S.M., Vashi N., Justice M.J. (2018). Rett syndrome: A neurological disorder with metabolic components. Open Biol..

[163] Qian J., Guan X., Xie B., Xu C., Niu J., Tang X., Li C.H., Colecraft H.M., Jaenisch R., Liu X.S. (2023). Multiplex epigenome editing of *MECP2* to rescue Rett syndrome neurons. Sci. Transl. Med..

[164] Kang J.G., Park J.S., Ko J.-H., Kim Y.-S. (2019). Regulation of gene expression by altered promoter methylation using a CRISPR/Cas9-mediated epigenetic editing system. Sci. Rep..

[165] Marx N., Grünwald-Gruber C., Bydlinski N., Dhiman H., Nguyen L.N., Klanert G., Borth N. (2018). CRISPR-Based Targeted Epigenetic Editing Enables Gene Expression Modulation of the Silenced Beta-Galactoside Alpha-2,6-Sialyltransferase 1 in CHO Cells. Biotechnol. J..

[166] Hanzawa N., Hashimoto K., Yuan X., Kawahori K., Tsujimoto K., Hamaguchi M., Tanaka T., Nagaoka Y., Nishina H., Morita S. (2020). Targeted DNA demethylation of the Fgf21 promoter by CRISPR/dCas9-mediated epigenome editing. Sci. Rep..

[167] Horii T., Morita S., Hino S., Kimura M., Hino Y., Kogo H., Nakao M., Hatada I. (2020). Successful generation of epigenetic disease model mice by targeted demethylation of the epigenome. Genome Biol..

[168] Noack F., Pataskar A., Schneider M., Buchholz F., Tiwari V.K., Calegari F. (2019). Assessment and site-specific manipulation of DNA (hydroxy-)methylation during mouse corticogenesis. Life Sci. Alliance.

[169] Liu R., Lang Z. (2020). The mechanism and function of active DNA demethylation in plants. J. Integr. Plant Biol..

[170] Parrilla-Doblas J.T., Ariza R.R., Roldán-Arjona T. (2017). Targeted DNA demethylation in human cells by fusion of a plant 5-methylcytosine DNA glycosylase to a sequence-specific DNA binding domain. Epigenetics.

[171] Hilton I.B., D’ippolito A.M., Vockley C.M., Thakore P.I., Crawford G.E., Reddy T.E., Gersbach C.A. (2015). Epigenome editing by a CRISPR-Cas9-based acetyltransferase activates genes from promoters and enhancers. Nat. Biotechnol..

[172] Cho H.-S., Kang J.G., Lee J.-H., Lee J.-J., Jeon S.K., Ko J.-H., Kim D.-S., Park K.-H., Kim Y.-S., Kim N.-S. (2015). Direct regulation of E-cadherin by targeted histone methylation of TALE-SET fusion protein in cancer cells. Oncotarget.

[173] Honarmand A., Mayall R., George I., Oberding L., Dastidar H., Fegan J., Chaudhuri S., Dole J., Feng S., Hoang D. (2014). A Multiplexed Transcription Activator-like Effector System for Detecting Specific DNA Sequences. ACS Synth. Biol..

[174] Sanjana N., Cong L., Zhou Y., Cunniff M.M., Feng G., Zhang F. (2012). A transcription activator-like effector toolbox for genome engineering. Nat. Protoc..

[175] Garcia-Bloj B., Moses C., Sgro A., Plani-Lam J., Arooj M., Duffy C., Thiruvengadam S., Sorolla A., Rashwan R., Mancera R.L. (2016). Waking up dormant tumor suppressor genes with zinc fingers, TALEs and the CRISPR/dCas9 system. Oncotarget.

[176] Chou C.-C., Lou Y.-C., Tang T.K., Chen C. (2010). Structure and DNA binding characteristics of the three-Cys2His2 domain of mouse testis zinc finger protein. Proteins Struct. Funct. Bioinform..

[177] Huber P.W., Morii T., Mei H.Y., Barton J.K. (1991). Structural polymorphism in the major groove of a 5S RNA gene complements the zinc finger domains of transcription factor IIIA. Proc. Natl. Acad. Sci. USA.

[178] Mandell J.G., Barbas C.F. (2006). Zinc Finger Tools: Custom DNA-binding domains for transcription factors and nucleases. Nucleic Acids Res..

[179] Urnov F.D., Miller J.C., Lee Y.-L., Beausejour C.M., Rock J.M., Augustus S., Jamieson A.C., Porteus M.H., Gregory P.D., Holmes M.C. (2005). Highly efficient endogenous human gene correction using designed zinc-finger nucleases. Nature.

[180] Lim W.F., Burdach J., Funnell A.P., Pearson R.C., Quinlan K.G., Crossley M. (2015). Directing an artificial zinc finger protein to new targets by fusion to a non-DNA-binding domain. Nucleic Acids Res..

[181] Liu Y., Zhang B., Kuang H., Korakavi G., Lu L.-Y., Yu X. (2016). Zinc Finger Protein 618 Regulates the Function of UHRF2 (Ubiquitin-like with PHD and Ring Finger Domains 2) as a Specific 5-Hydroxymethylcytosine Reader. J. Biol. Chem..

[182] Sander J.D., Maeder M.L., Reyon D., Voytas D.F., Joung J.K., Dobbs D. (2010). ZiFiT (Zinc Finger Targeter): An updated zinc finger engineering tool. Nucleic Acids Res..

[183] Maeder M.L., Thibodeau-Beganny S., Sander J.D., Voytas D.F., Joung J.K. (2009). Oligomerized pool engineering (OPEN): An ‘open-source’ protocol for making customized zinc-finger arrays. Nat. Protoc..

[184] Sander J.D., Dahlborg E.J., Goodwin M.J., Cade L., Zhang F., Cifuentes D., Curtin S.J., Blackburn J., Thibodeau-Beganny S., Qi Y. (2010). Selection-free zinc-finger-nuclease engineering by context-dependent assembly (CoDA). Nat. Methods.

[185] Yee J.-K. (2016). Off-target effects of engineered nucleases. FEBS J..

[186] Rogers J.M., Barrera L.A., Reyon D., Sander J.D., Kellis M., Joung J.K., Bulyk M.L. (2015). Context influences on TALE–DNA binding revealed by quantitative profiling. Nat. Commun..

[187] Nitsch S., Mussolino C. (2018). Generation of TALE-Based Designer Epigenome Modifiers. Epigenome Ed. Methods Protoc..

[188] Waryah C.B., Moses C., Arooj M., Blancafort P. (2018). Zinc Fingers, TALEs, and CRISPR Systems: A Comparison of Tools for Epigenome Editing. Epigenome Ed. Methods Protoc..

[189] Sung C.K., Yim H. (2020). CRISPR-mediated promoter de/methylation technologies for gene regulation. Arch. Pharmacal Res..

[190] Nguyen T.V., Lister R. (2021). Genomic targeting of TET activity for targeted demethylation using CRISPR/Cas9. Methods Mol. Biol..

[191] Sapozhnikov D.M., Szyf M. (2021). Unraveling the functional role of DNA demethylation at specific promoters by targeted steric blockage of DNA methyltransferase with CRISPR/dCas9. Nat. Commun..

[192] Xu X., Tao Y., Gao X., Zhang L., Li X., Zou W., Ruan K., Wang F., Xu G.-L., Hu R. (2016). A CRISPR-based approach for targeted DNA demethylation. Cell Discov..

[193] Cheng A.W., Jillette N., Lee P., Plaskon D., Fujiwara Y., Wang W., Taghbalout A., Wang H. (2016). Casilio: A versatile CRISPR-Cas9-Pumilio hybrid for gene regulation and genomic labeling. Cell Res..

[194] Guo J., Gaj T., Barbas C.F. (2010). Directed Evolution of an Enhanced and Highly Efficient FokI Cleavage Domain for Zinc Finger Nucleases. J. Mol. Biol..

[195] Li X., Wang Z., Huang J., Luo H., Zhu S., Yi H., Zheng L., Hu B., Yu L., Li L. (2018). Specific zinc finger-induced methylation of PD-L1 promoter inhibits its expression. FEBS Open Bio.

[196] Bennett C. Are Zinc Finger Nucleases Making a Comeback? 30 November 2017 Genetic Engineering & Biotechnology News. https://www.genengnews.com/insights/are-zinc-finger-nucleases-making-a-comeback/.

[197] Lanio T., Jeltsch A., Pingoud A. (1998). Towards the design of rare cutting restriction endonucleases: Using directed evolution to generate variants of EcoRV differing in their substrate specificity by two orders of magnitude. J. Mol. Biol..

[198] Lei Y., Zhang X., Su J., Jeong M., Gundry M.C., Huang Y.-H., Zhou Y., Li W., Goodell M.A. (2017). Targeted DNA methylation in vivo using an engineered dCas9-MQ1 fusion protein. Nat. Commun..

[199] Li R., Meng Q., Qi J., Hu L., Huang J., Zhang Y., Yang J., Sun J. (2022). Microinjection-based CRISPR/Cas9 mutagenesis in the decapoda crustaceans *Neocaridina heteropoda* and *Eriocheir sinensis*. J. Exp. Biol..

[200] Laustsen A., Bak R.O. (2019). Electroporation-Based CRISPR/Cas9 Gene Editing Using Cas9 Protein and Chemically Modified sgRNAs. CRISPR Gene Ed. Methods Protoc..

[201] Danthinne X., Imperiale M.J. (2000). Production of first generation adenovirus vectors: A review. Gene Ther..

[202] Rice S.A., Klessig D.F., Williams J. (1987). Multiple effects of the 72-kDa, adenovirus-specified DNA binding protein on the efficiency of cellular transformation. Virology.

[203] Zhang Y., Schneider R.J. (1994). Adenovirus inhibition of cell translation facilitates release of virus particles and enhances degradation of the cytokeratin network. J. Virol..

[204] Yang Y., Ertl H.C., Wilson J.M. (1994). MHC class I-cestricted cytotoxic T lymphocytes to viral antigens destroy hepatocytes in mice infected with E1-deleted recombinant adenoviruses. Immunity.

[205] Jerome S. (2005). Adenovirus vectors deleted for genes essential for viral DNA replication. Front. Biosci..

[206] Cannon P., June C.H. (2011). Chemokine receptor 5 knockout strategies. Curr. Opin. HIV AIDS.

[207] Holkers M., de Vries A.A.F., Gonçalves M.A.F.V. (2012). Nonspaced inverted DNA repeats are preferential targets for homology-directed gene repair in mammalian cells. Nucleic Acids Res..

[208] Hu Z., Ding W., Zhu D., Yu L., Jiang X., Wang X., Zhang C., Wang L., Ji T., Liu D. (2014). TALEN-mediated targeting of HPV oncogenes ameliorates HPV-related cervical malignancy. J. Clin. Investig..

[209] Krasnykh V., Dmitriev I., Navarro J.G., Belousova N., Kashentseva E., Xiang J., Douglas J.T., Curiel D.T. (2000). Advanced generation adenoviral vectors possess augmented gene transfer efficiency based upon coxsackie adenovirus receptor-independent cellular entry capacity. Cancer Res..

[210] Hensen L.C., Hoeben R.C., Bots S.T. (2020). Adenovirus Receptor Expression in Cancer and Its Multifaceted Role in Oncolytic Adenovirus Therapy. Int. J. Mol. Sci..

[211] Lowenstein P.R., Mandel R.J., Xiong W.-D., Kroeger K., Castro M.G. (2007). Immune responses to adenovirus and adeno-associated vectors used for gene therapy of brain diseases: The role of immunological synapses in understanding the cell biology of neuroimmune interactions. Curr. Gene Ther..

[212] Vannucci L., Lai M., Chiuppesi F., Ceccherini-Nelli L., Pistello M. (2013). Viral vectors: A look back and ahead on gene transfer technology. New Microbiol..

[213] Anguela X.M., Sharma R., Doyon Y., Miller J.C., Li H., Haurigot V.A., Rohde M.E., Wong S.Y., Davidson R.J., Zhou S. (2013). Robust ZFN-mediated genome editing in adult hemophilic mice. Blood.

[214] Händel E.-M., Gellhaus K., Khan K., Bednarski C., Cornu T.I., Müller-Lerch F., Kotin R.M., Heilbronn R., Cathomen T. (2012). Versatile and Efficient Genome Editing in Human Cells by Combining Zinc-Finger Nucleases With Adeno-Associated Viral Vectors. Hum. Gene Ther..

[215] Ran F.A., Cong L., Yan W.X., Scott D.A., Gootenberg J.S., Kriz A.J., Zetsche B., Shalem O., Wu X., Makarova K.S. (2015). In vivo genome editing using Staphylococcus aureus Cas9. Nature.

[216] Rahman S.H., Bobis-Wozowicz S., Chatterjee D., Gellhaus K., Pars K., Heilbronn R., Jacobs R., Cathomen T. (2013). The Nontoxic Cell Cycle Modulator Indirubin Augments Transduction of Adeno-Associated Viral Vectors and Zinc-Finger Nuclease-Mediated Gene Targeting. Hum. Gene Ther..

[217] Swiech L., Heidenreich M., Banerjee A., Habib N., Li Y., Trombetta J.J., Sur M., Zhang F. (2014). In vivo interrogation of gene function in the mammalian brain using CRISPR-Cas9. Nat. Biotechnol..

[218] Tabebordbar M., Zhu K., Cheng J.K.W., Chew W.L., Widrick J.J., Yan W.X., Maesner C., Wu E.Y., Xiao R., Ran F.A. (2016). In vivo gene editing in dystrophic mouse muscle and muscle stem cells. Science.

[219] Zabaleta N., Dai W., Bhatt U., Hérate C., Maisonnasse P., Chichester J.A., Sanmiguel J., Estelien R., Michalson K.T., Diop C. (2021). An AAV-based, room-temperature-stable, single-dose COVID-19 vaccine provides durable immunogenicity and protection in non-human primates. Cell Host Microbe.

[220] Zhao S., Ke J., Yang B., Tan F., Yang J., Lin C.-P., Wang H., Zhong G. (2022). A protective AAV vaccine for SARS-CoV-2. Signal Transduct. Target. Ther..

[221] Narayan O., Zink M., Huso D., Sheffer D., Crane S., Kennedy-Stoskopf S., Jolly P., Clements J. (1988). Lentiviruses of animals are biological models of the human immunodeficiency viruses. Microb. Pathog..

[222] Sakuma T., Barry M.A., Ikeda Y. (2012). Lentiviral vectors: Basic to translational. Biochem. J..

[223] Thomas C.E., Ehrhardt A., Kay M.A. (2003). Progress and problems with the use of viral vectors for gene therapy. Nat. Rev. Genet..

[224] Mátrai J., Chuah M.K., Vandendriessche T. (2010). Recent Advances in Lentiviral Vector Development and Applications. Mol. Ther..

[225] Lombardo A.L., Genovese P., Beausejour C.M., Colleoni S., Lee Y.-L., A Kim K., Ando D., Urnov F.D., Galli C., Gregory P. (2007). Gene editing in human stem cells using zinc finger nucleases and integrase-defective lentiviral vector delivery. Nat. Biotechnol..

[226] Apolonia L., Waddington S., Fernandes C., Ward N.J., Bouma G., Blundell M., Thrasher A.J., Collins M.K., Philpott N.J. (2007). Stable Gene Transfer to Muscle Using Non-integrating Lentiviral Vectors. Mol. Ther..

[227] Nayak S., Herzog R.W. (2010). Progress and prospects: Immune responses to viral vectors. Gene Ther..

[228] Zhou H.-S., Liu D.-P., Liang C.-C. (2004). Challenges and strategies: The immune responses in gene therapy. Med. Res. Rev..

[229] Nair V. (2008). Retrovirus-induced oncogenesis and safety of retroviral vectors. Curr. Opin. Mol. Ther..

[230] Baum C., Kustikova O., Modlich U., Li Z., Fehse B. (2006). Mutagenesis and Oncogenesis by Chromosomal Insertion of Gene Transfer Vectors. Hum. Gene Ther..

[231] Themis M., Waddington S.N., Schmidt M., von Kalle C., Wang Y., Al-Allaf F., Gregory L.G., Nivsarkar M., Themis M., Holder M.V. (2005). Oncogenesis Following Delivery of a Nonprimate Lentiviral Gene Therapy Vector to Fetal and Neonatal Mice. Mol. Ther..

[232] Baum C., Düllmann J., Li Z., Fehse B., Meyer J., Williams D.A., Von Kalle C. (2003). Side effects of retroviral gene transfer into hematopoietic stem cells. Blood.

[233] Modlich U., Kustikova O.S., Schmidt M., Rudolph C., Meyer J., Li Z., Kamino K., Von Neuhoff N., Schlegelberger B., Kuehlcke K. (2005). Leukemias following retroviral transfer of multidrug resistance 1 (MDR1) are driven by combinatorial insertional mutagenesis. Blood.

[234] Nowrouzi A., Glimm H., Von Kalle C., Schmidt M. (2011). Retroviral Vectors: Post Entry Events and Genomic Alterations. Viruses.

[235] Baum C., von Kalle C., Staal F.J., Li Z., Fehse B., Schmidt M., Weerkamp F., Karlsson S., Wagemaker G., A Williams D. (2004). Chance or necessity? Insertional Mutagenesis in Gene Therapy and Its Consequences. Mol. Ther..

[236] Jacobsen L.B., Calvin S.A., Lobenhofer E.K. (2009). Transcriptional effects of transfection: The potential for misinterpretation of gene expression data generated from transiently transfected cells. Biotechniques.

[237] Capretto L., Carugo D., Mazzitelli S., Nastruzzi C., Zhang X. (2013). Microfluidic and lab-on-a-chip preparation routes for organic nanoparticles and vesicular systems for nanomedicine applications. Adv. Drug Deliv. Rev..

[238] Rawal S., Patel M.M. (2019). Threatening cancer with nanoparticle aided combination oncotherapy. J. Control. Release.

[239] Shoari A., Tooyserkani R., Tahmasebi M., Löwik D.W.P.M. (2021). Delivery of Various Cargos into Cancer Cells and Tissues via Cell-Penetrating Peptides: A Review of the Last Decade. Pharmaceutics.

[240] de Ilarduya C.T., Sun Y., Düzgüneş N. (2010). Gene delivery by lipoplexes and polyplexes. Eur. J. Pharm. Sci..

[241] Zu H., Gao D. (2021). Non-viral Vectors in Gene Therapy: Recent Development, Challenges, and Prospects. AAPS J..

[242] Obika S., Yu W., Shimoyama A., Uneda T., Minami T., Miyashita K., Doi T., Imanishi T. (1999). Properties of Cationic Liposomes Composed of Cationic Lipid YKS-220 Having an Ester Linkage: Adequate Stability, High Transfection Efficiency, and Low Cytotoxicity. Biol. Pharm. Bull..

[243] Almofti M.R., Harashima H., Shinohara Y., Almofti A., Baba Y., Kiwada H. (2003). Cationic liposome-mediated gene delivery: Biophysical study and mechanism of internalization. Arch. Biochem. Biophys..

[244] Conde J., Bao C., Tan Y., Cui D., Edelman E.R., Azevedo H.S., Byrne H.J., Artzi N., Tian F. (2015). Dual Targeted Immunotherapy via In Vivo Delivery of Biohybrid RNAi-Peptide Nanoparticles to Tumor-Associated Macrophages and Cancer Cells. Adv. Funct. Mater..

[245] Yuan Y., Xu L., Dai S., Wang M., Wang H. (2017). A facile supramolecular approach to fabricate multifunctional upconversion nanoparticles as a versatile platform for drug loading, in vivo delivery and tumor imaging. J. Mater. Chem. B.

[246] Wan X., Sun R., Bao Y., Zhang C., Wu Y., Gong Y. (2021). In Vivo Delivery of siRNAs Targeting EGFR and BRD4 Expression by Peptide-Modified Redox Responsive PEG–PEI Nanoparticles for the Treatment of Triple-Negative Breast Cancer. Mol. Pharm..

[247] Han J.P., Kim M., Choi B.S., Lee J.H., Lee G.S., Jeong M., Lee Y., Kim E.-A., Oh H.-K., Go N. (2022). In vivo delivery of CRISPR-Cas9 using lipid nanoparticles enables antithrombin gene editing for sustainable hemophilia A and B therapy. Sci. Adv..

[248] Lokugamage M.P., Vanover D., Beyersdorf J., Hatit M.Z.C., Rotolo L., Echeverri E.S., Peck H.E., Ni H., Yoon J.-K., Kim Y. (2021). Optimization of lipid nanoparticles for the delivery of nebulized therapeutic mRNA to the lungs. Nat. Biomed. Eng..

[249] Kim J., Jozic A., Lin Y., Eygeris Y., Bloom E., Tan X., Acosta C., MacDonald K.D., Welsher K.D., Sahay G. (2022). Engineering Lipid Nanoparticles for Enhanced Intracellular Delivery of mRNA through Inhalation. ACS Nano.

[250] Rotolo L., Vanover D., Bruno N.C., Peck H.E., Zurla C., Murray J., Noel R.K., O’farrell L., Araínga M., Orr-Burks N. (2022). Species-agnostic polymeric formulations for inhalable messenger RNA delivery to the lung. Nat. Mater..

[251] Pei Y., Bao Y., Sacchetti C., Brady J., Gillard K., Yu H., Roberts S., Rajappan K., Tanis S.P., Perez-Garcia C.G. (2022). Synthesis and bioactivity of readily hydrolysable novel cationic lipids for potential lung delivery application of mRNAs. Chem. Phys. Lipids.

[252] Tam A., Kulkarni J., An K., Li L., Dorscheid, Singhera G., Bernatchez P., Reid G., Chan K., Witzigmann D. (2022). Lipid nanoparticle formulations for optimal RNA-based topical delivery to murine airways. Eur. J. Pharm. Sci..

[253] Jin H., Jeong M., Lee G., Kim M., Yoo Y., Kim H.J., Cho J., Lee Y., Lee H. (2022). Engineered Lipid Nanoparticles for the Treatment of Pulmonary Fibrosis by Regulating Epithelial-Mesenchymal Transition in the Lungs. Adv. Funct. Mater..

[254] Wadhwa A., Aljabbari A., Lokras A., Foged C., Thakur A. (2020). Opportunities and Challenges in the Delivery of mRNA-Based Vaccines. Pharmaceutics.

[255] Gaj T., Guo J., Kato Y., Sirk S.J., Barbas C.F. (2012). Targeted gene knockout by direct delivery of zinc-finger nuclease proteins. Nat. Methods.

[256] Rádis-Baptista G., Campelo I.S., Morlighem J.R., Melo L.M., Freitas V.J. (2017). Cell-penetrating peptides (CPPs): From delivery of nucleic acids and antigens to transduction of engineered nucleases for application in transgenesis. J. Biotechnol..

[257] Gagat M., Zielińska W., Grzanka A. (2017). Cell-penetrating peptides and their utility in genome function modifications (Review). Int. J. Mol. Med..

[258] Wang Q., Yu J., Kadungure T., Beyene J., Zhang H., Lu Q. (2018). ARMMs as a versatile platform for intracellular delivery of macromolecules. Nat. Commun..

[259] Wang Q., Lu Q. (2017). Plasma membrane-derived extracellular microvesicles mediate non-canonical intercellular NOTCH signaling. Nat. Commun..

[260] Ramakrishna S., Kim Y.-H., Kim H. (2013). Stability of Zinc Finger Nuclease Protein Is Enhanced by the Proteasome Inhibitor MG132. PLoS ONE.

[261] Lo C.-L., Choudhury S.R., Irudayaraj J., Zhou F.C. (2017). Epigenetic Editing of Ascl1 Gene in Neural Stem Cells by Optogenetics. Sci. Rep..

[262] Huisman C., van der Wijst M., Schokker M., Blancafort P., Terpstra M.M., Kok K., van der Zee A.G.J., Schuuring E., A Wisman G.B., Rots M.G. (2016). Re-expression of Selected Epigenetically Silenced Candidate Tumor Suppressor Genes in Cervical Cancer by TET2-directed Demethylation. Mol. Ther..

[263] Gallego-Bartolome J., Gardiner J., Liu W., Papikian A., Ghoshal B., Kuo H.Y., Zhao J.M.-C., Segal D.J., Jacobsen S.E. (2018). Targeted DNA demethylation of the Arabidopsis genome using the human TET1 catalytic domain. Proc. Natl. Acad. Sci. USA.

[264] Choudhury S.R., Cui Y., Lubecka K., Stefanska B., Irudayaraj J. (2016). CRISPR-dCas9 mediated TET1 targeting for selective DNA demethylation at *BRCA1* promoter. Oncotarget.

[265] Wang M., He L., Chen B., Wang Y., Wang L., Zhou W., Zhang T., Cao L., Zhang P., Xie L. (2022). Transgenerationally Transmitted DNA Demethylation of a Spontaneous Epialleles Using CRISPR/dCas9-TET1cd Targeted Epigenetic Editing in Arabidopsis. Int. J. Mol. Sci..

